# Coevolution, Dynamics and Allostery Conspire in Shaping Cooperative Binding and Signal Transmission of the SARS-CoV-2 Spike Protein with Human Angiotensin-Converting Enzyme 2

**DOI:** 10.3390/ijms21218268

**Published:** 2020-11-04

**Authors:** Gennady Verkhivker

**Affiliations:** 1 Graduate Program in Computational and Data Sciences, Schmid College of Science and Technology, Chapman University, Orange, CA 92866, USA; verkhivk@chapman.edu; Tel.: +1-714-516-4586; 2 Department of Biomedical and Pharmaceutical Sciences, Chapman University School of Pharmacy, Irvine, CA 92618, USA

**Keywords:** SARS-CoV spike protein, ACE2, coevolution, molecular dynamics, alanine scanning, binding free energy, allosteric interactions, signal transmission

## Abstract

Binding to the host receptor is a critical initial step for the coronavirus SARS-CoV-2 spike protein to enter into target cells and trigger virus transmission. A detailed dynamic and energetic view of the binding mechanisms underlying virus entry is not fully understood and the consensus around the molecular origins behind binding preferences of SARS-CoV-2 for binding with the angiotensin-converting enzyme 2 (ACE2) host receptor is yet to be established. In this work, we performed a comprehensive computational investigation in which sequence analysis and modeling of coevolutionary networks are combined with atomistic molecular simulations and comparative binding free energy analysis of the SARS-CoV and SARS-CoV-2 spike protein receptor binding domains with the ACE2 host receptor. Different from other computational studies, we systematically examine the molecular and energetic determinants of the binding mechanisms between SARS-CoV-2 and ACE2 proteins through the lens of coevolution, conformational dynamics, and allosteric interactions that conspire to drive binding interactions and signal transmission. Conformational dynamics analysis revealed the important differences in mobility of the binding interfaces for the SARS-CoV-2 spike protein that are not confined to several binding hotspots, but instead are broadly distributed across many interface residues. Through coevolutionary network analysis and dynamics-based alanine scanning, we established linkages between the binding energy hotspots and potential regulators and carriers of signal communication in the virus–host receptor complexes. The results of this study detailed a binding mechanism in which the energetics of the SARS-CoV-2 association with ACE2 may be determined by cumulative changes of a number of residues distributed across the entire binding interface. The central findings of this study are consistent with structural and biochemical data and highlight drug discovery challenges of inhibiting large and adaptive protein–protein interfaces responsible for virus entry and infection transmission.

## 1. Introduction

**Figure 1:**
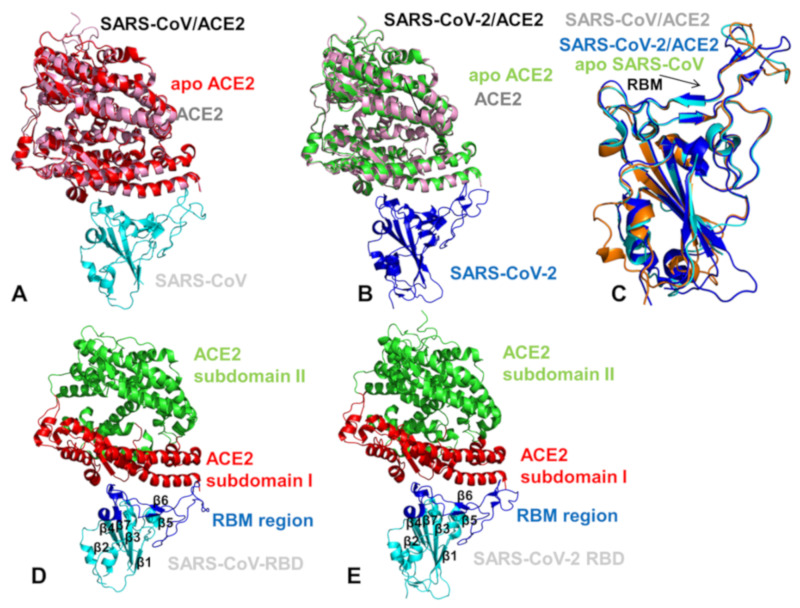
Structural organization of the SARS-CoV-RBD (severe acute respiratory syndrome coronavirus receptor binding domain) and SARS-CoV-2-RBD (severe acute respiratory syndrome coronavirus 2 receptor binding domain) complexes with human ACE enzyme. (**A**) A general overview of the SARS-CoV-RBD complex with ACE2 (pdb id 2AJF). The SARS-CoV-RBD is in cyan ribbons and the bound ACE2 enzyme is in pink ribbons. The crystal structure of the unbound native human ACE2 enzyme (pdb id 1R42) is superimposed and shown in red ribbons. (**B**) A general overview of the SARS-CoV-2-RBD complex with ACE2 (pdb id 6M0J). The SARS-CoV-2-RBD is shown in blue ribbons, the bound ACE2 enzyme is in pink ribbons, and the unbound form of ACE2 is in green. (**C**) Structural superposition of the unbound SARS-CoV-RBD (pdb id 2GHV) (in orange), bound SARS-CoV-RBD (pdb id 2AJF) (in cyan), and SARS-CoV-2-RBD (pdb id 6M0J) (in blue). Structural similarity of the RBM motif is indicated by an arrow and annotated. A moderate local mobility of the flexible ridge loop at the tip of the RBM in the unbound and bound SARS-CoV-RBD forms is evident. (**D**) A general overview of the secondary structure elements and binding interface in the SARS-CoV-RBD complex with human ACE2. The SARS-CoV-RBD is shown in cyan and secondary structure elements are annotated. The RBM region in SARS-CoV-RBD (residues 424–494) that provides the contact interface with ACE2 is highlighted in blue ribbons and annotated. The subdomain I of human ACE2 is shown in red ribbons and the subdomain II is shown in green ribbons. (**E**) A general overview of the secondary structure elements and binding interface in the SARS-CoV-2 RBD complex with human ACE2. The SARS-CoV-RBD is shown in cyan and secondary structure elements are annotated. The RBM region is in blue ribbons and annotated. The subdomains I and II of human ACE2 are shown in red and green ribbons, respectively.

**Figure 2:**
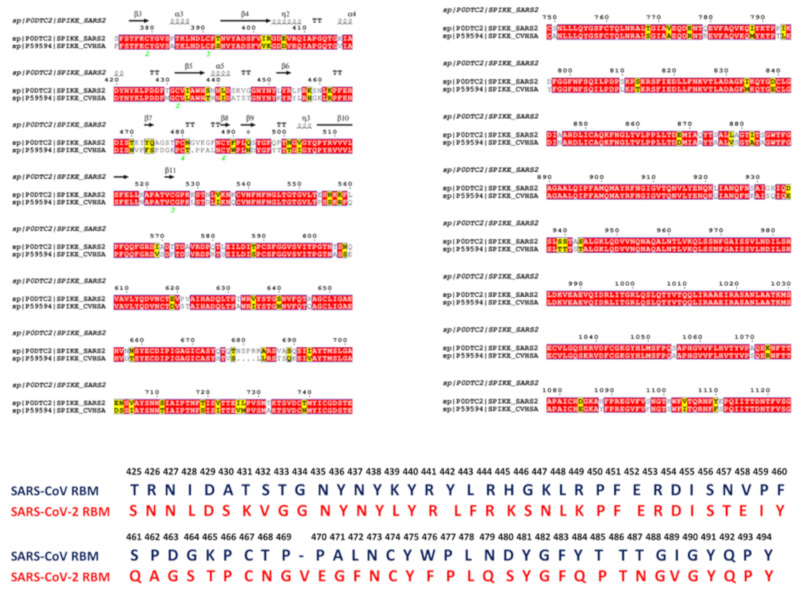
Sequence alignment of the SARS-CoV and SARS-CoV-2 RBD proteins. (Top panel) The sequence alignment of the SARS-CoV and SARS-CoV-2 spike proteins. (Bottom panel). The sequence comparison of the RBM residues. SARS-CoV-RBD residues are in blue and SARS-CoV-2 RBD residues are in red. The close-up of the RBM alignment highlights presence of evolutionary conserved positions even in the most variable interface region. The emergence of a significant number of amino acid modifications between the SARS-CoV and SARS-CoV-2 RBD proteins can be also seen.

The coronavirus disease 2019 (COVID-19) pandemic has emerged as a global international health crisis that has spread over the world with far-reaching implications for global economy, peace, and security [1,2]. The coronavirus SARS-CoV-2 (previously known as nCoV-19) is associated with the acute respiratory distress syndrome [1,2] and is similar to the severe acute respiratory syndrome (SARS) and Middle East respiratory syndrome (MERS) viruses [3]. The genomic sequences of the coronavirus SARS-CoV-2 showed a high level of sequence similarity between the SARS-CoV-2, SARS, and MERS proteins involved in the replication cycle [4,5,6]. SARS-CoV-2 has four main structural proteins: spike (S) glycoprotein, small envelope (E) glycoprotein, membrane (M) glycoprotein, and nucleocapsid (N) protein, along with several accessory proteins [7,8,9]. Recent studies have identified that SARS-CoV-2 uses ACE2 enzyme [10,11,12] and the cellular protease transmembrane protease serine 2 (TMPRSS2) as cell entry receptors. SARS-CoV-2 binds to the ACE2 receptor on the surface of the host cell using binding of the S1 region of the virus spike (S) protein followed by the fusion of the viral and cellular membranes mediated by the S2 subunit of the spike S protein [13]. The crystal structure of the receptor-binding domain (RBD) of SARS-CoV-2 spike protein in complex with human ACE2 revealed a binding mode similar to the one observed for SARS-CoV spike protein [14]. Furthermore, the real-time surface plasmon resonance (SPR) assays showed that the interactions between SARS-CoV-2-RBD and human ACE2 are specific, leading to the four-fold stronger binding affinity as compared with the SARS-CoV-RBD [14]. The initial comparative analyses based on decade-long structural studies of SARS coronavirus established the key interactions between SARS-CoV-RBD and its host receptor ACE2 enzyme that may regulate both the cross-species and human-to-human transmissions [15]. This study suggested that SARS-CoV-2 (year 2019) likely uses human ACE2 more efficiently than human SARS-CoV (year 2003). Furthermore, this analysis reinforced the notion that the mechanism of binding energetics with ACE2 may be linked and ultimately determine SARS infectivity, indicating that SARS-CoV-2 may have evolved its capability for the stronger binding with human ACE2 enzyme and accordingly may have developed a unique capacity for rapid infection transmission among humans. Although binding of SARS-CoV-RBD to the ACE2 receptor is now recognized as a critical initial step for virus entry into target cells, the molecular determinants of binding specificity for SARS-CoV-2 are not fully established, owing to structurally similar binding interfaces shared by these proteins with human ACE2. Recent studies elaborated functional mechanisms of ACE2 in mediating entry of SARS-CoV and SARS-CoV-2 spike proteins [16,17,18,19,20]. SARS spike proteins are trimeric fusion proteins with two main domains, the S1 and the S2 domain. The cryo-EM structures of the SARS-CoV-2 spike ectodomain trimer in two distinct conformations provided evidence of large conformational changes of the SARS-CoV-2 spike glycoprotein and identified conserved and accessible epitopes across S glycoproteins confirming that SARS-CoV-2 and SARS-CoV bind with similar affinities to human ACE2 [16]. The cryo-EM structures of MERS-CoV and SARS-CoV spike glycoproteins further characterized the dynamic principles of receptor binding, showing that during binding with the host receptor, the RBD of S1 trimer undergoes hinge motions between receptor-inaccessible “down” and the receptor-accessible “up” conformations [17,18,19]. The recent cryo-EM structure of the SARS-CoV-2 trimeric spike in the prefusion conformation allowed for reconstruction of an asymmetrical trimer in which a single RBD was observed only in the up conformation [20]. This study also employed biophysical analysis and binding assays to demonstrate that in this conformation the SARS-CoV-2 trimeric spike protein binds at least 10 times stronger to the common host cell receptor than the SARS-CoV protein [20]. The crystal structures of the SARS-CoV-RBD [21,22,23] and SARS-CoV-2-RBD proteins bound to the host receptor ACE2 [24] (Figure 1) provided a foundation for understanding dynamic and energetic differences between these spike proteins. These studies reported a high degree of structural similarity between SARS-CoV-RBD and SARS-CoV-2-RBD proteins with a root mean square deviation, RMSD = 1.2 Å for Cα atoms, and even a lower RMSD = 0.68 Å between ACE2 conformations, also indicating that the structural arrangement of the binding interfaces in the SARS-CoV-RBD and SARS-CoV-2-RBD complexes with ACE2 is virtually identical [24]. According to the crystallographic data, even in the more variable receptor binding motif (RBM) of the binding interface, SARS-CoV-RBD and SARS-CoV-2-RBD maintain significant structurally similarity (RMSD = 1.3–1.4 Å) showing a visible local conformational change only in the remote end of the RBM region [24]. Sequence analysis also revealed a considerable similarity between SARS-CoV-2 and SARS-CoV RBD proteins (Figure 2), suggesting convergent evolution of spike structural folds for binding to ACE2 enzyme [21,24].

The observed sequence similarities are evident even in the variable RBM interface region where many identical residues are shared between SARS-CoV-RBD and SARS-CoV-2-RBD proteins (Figure 2). However, a number of residues at the binding interface with ACE2 are different between SARS-CoV-RBD and SARS-CoV-2 RBD proteins (Table S1). Another crystal structure of the RBD of the ACE2 complex with the engineered chimera of spike protein of SARS-CoV-2 was recently reported that retained the core from the SARS-CoV-RBD as the crystallization scaffold and used the RBM from SARS-CoV-2 as the functionally relevant motif [25]. The structure of this chimeric RBD–ACE2 complex is highly similar to the structure of the SARS-CoV-2 wild-type RBD–ACE2 complex [24]. Importantly, both the chimeric and native SARS-CoV-2 RBD proteins displayed significantly stronger binding affinities with ACE2 as compared with the SARS-CoV RBD. It was conjectured that the RBM region in the SARS-CoV-2-RBD has a more compact conformation and features several modifications that may contribute to stabilization of the binding interface hotspots and explain the stronger binding with ACE2 [25].

**Figure 3:**
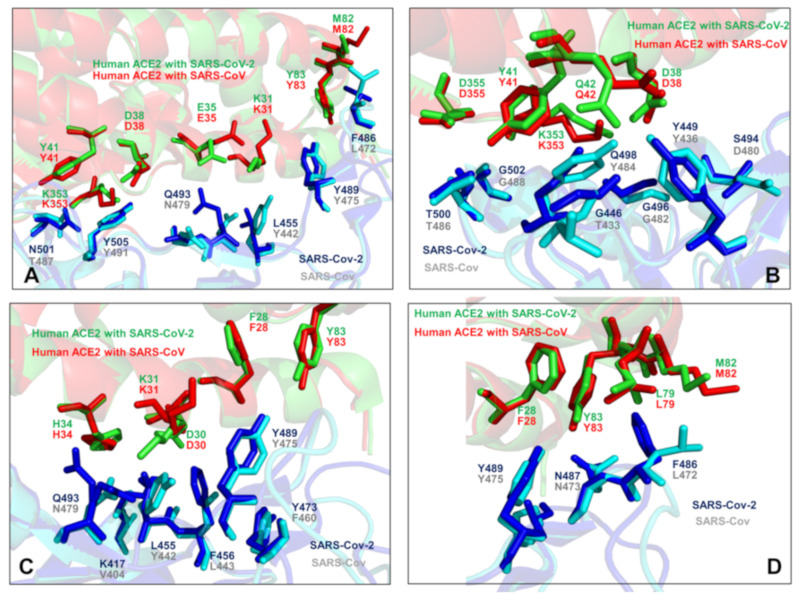
An overview of the binding interface residues in the SARS-CoV-RBD and SARS-CoV-2-RBD complexes with human ACE enzyme. The minimized average equilibrium structures obtained from MD simulations are used to depict the binding interface regions and highlight small conformational changes. (**A**) The N-terminus segment of the interface is shown. The SARS-CoV-RBD residues T487, Y491, N479, Y442, Y475, and L472 from the complex with ACE2 are shown in cyan sticks. The corresponding SARS-CoV-2-RBD residues N501, Y505, Q493, L455, Y489, and F486 are shown in blue sticks. (**B**) Another binding interface region is shown with SARS-CoV-RBD residues T486, G488, T433, G482, Y484, Y436, and D480 (cyan sticks). The corresponding SARS-CoV-2-RBD residues T500, G502, G446, G496, Q498, Y449, and S494 are shown in blue sticks. (**C**) The central segment of the binding interface with SARS-CoV-RBD residues N479, V404, Y442, L443, F460, and Y475 (cyan sticks). The SARS-CoV-2-RBD residues are Q493, K417, L455, F456, Y473, and Y489 (blue sticks). (**D**) The binding interface flexible ridge with SARS-CoV-RBD residues Y475, N473, and L472 (cyan sticks) and SARS-CoV-2-RBD residues Y489, N487, and F486 (in blue sticks). In all panels ACE2 interacting residues are annotated and shown in red sticks for the SARS-CoV-RBD complex and green sticks for the complex with SARS-CoV-2-RBD.

The cryo–electron microscopy structures of the full-length human ACE2 in the presence of the neutral amino acid transporter B^0^AT1 with or without the RBD of the spike glycoprotein of SARS-CoV-2 unveiled an elegant architecture suggesting simultaneous binding of two S protein trimers to an ACE2 homodimer [26]. The comparative analysis of the ACE2 binding interfaces with SARS-CoV-2 RBD and SARS-CoV RBD proteins suggested a complex balance of the interactions, where some contacts may strengthen binding of SARS-CoV-2-RBD while other changes could be counterproductive and weaken SARS-CoV-2-RBD binding [26]. These pioneering studies also indicated that the molecular and energetic determinants of SARS-CoV-2 selectivity are yet to be fully delineated and may involve a delicate balance of multiple thermodynamic factors that require a detailed biophysical analysis of the interplay between structure, dynamics, and binding. Combined, sequence, and structure analyses of these complexes structures [21,24,25,26] highlighted how sequence variations in the SARS-CoV-RBD and SARS-CoV-2-RBD proteins can be reflected in various local conformational changes of the binding interfaces with the ACE2 host receptor (Figure 3). The crystallographic studies and biophysical experiments of the SARS-CoV-2 protein binding with ACE2 concluded that binding affinity changes are highly relevant and critically important for SARS-CoV-2 selective entry to the host receptor. While the structure-based energetic changes can shed light onto the principles of the binding mechanism, the interplay of local and global dynamic variations and accompanied free energy changes need to be quantified to better explain the unusual selectivity of the SARS-CoV-2 virus entry [24,25,26]. The biochemical and mutagenesis studies explored energetics and binding mechanisms of SARS-CoV-2 interactions with the host receptor [27,28]. Deep mutational scanning of SARS-CoV-2 RBD revealed protein stability patterns and constraints on folding and ACE2 binding [27]. It was discovered that a surprisingly large number of amino acid modifications, including the binding interface residues, could be tolerated and even improve ACE2 binding.

In particular, mutations of several important interface positions can enhance ACE2 binding affinity (N501F, N501T, and Q498Y) [27] suggesting dynamic and energetic plasticity of the SARS-CoV-2 interaction network in which mutations that attenuate the polar nature of these residues may enhance binding affinity. Using deep mutagenesis, it was also demonstrated that human ACE2 is only suboptimal for binding of the SARS-CoV-2 RBD as ACE2 variants near the interface can result in the improved binding and simultaneously enhance folding stability [28]. These biochemical studies suggested that the protein and binding affinity of the SARS-CoV-2-RBD with ACE2 may involve a subtle cumulative effect of many small contributions from multiple positions that are broadly distributed across the intermolecular interface and in the protein core regions.

The rapidly emerging plethora of computational studies examined SARS-CoV-2-RBD interactions with ACE2 enzyme using the recent crystal structures [29,30,31,32,33,34,35,36,37]. It was proposed that SARS-CoV-2-RBD may have adopted a specific strategy for achieving favorable binding affinity by exploiting the larger binding interface as compared to the SARS-CoV-RBD that employs the fewer contacts and could rely only on major hotpot centers [29]. This study suggested that a more favorable binding affinity of SARS-CoV-2 RBD can be achieved through a combination of multiple interface contacts and protein stability enhancement. Microsecond molecular dynamics (MD) simulations examined the molecular determinants of the higher affinity of SARS-CoV-2 toward ACE2 by predicting the key role of Q498 and F486 residues (corresponding to Y484 and L472 in SARS-CoV) (Figure 3), also noticing that the electrostatic potential at the binding interface with the two SARS variants showed no appreciable differences [30]. This study proposed that the recognition loop of the RBM (residues 470–491 in SARS-CoV-2) and its persistent interactions with ACE2 may contribute to the enhanced binding affinity towards ACE2 and provide a molecular link with human-to-human transmissibility and virus infectivity. A related study also highlighted the role of F486 from SARS-CoV-2 RBM region that protrudes deeply into a hydrophobic pocket of ACE2 formed by F28, L79, Y83, and L97 residues (Figure 3C,D), which points to a potential role of the flexible loop 480-CNGVEGFNC-488 in modulating binding selectivity [31]. The latest comprehensive computational study employed molecular dynamics (MD) simulations to reveal a balance of hydrophobic interactions and elaborate hydrogen-bonding network in the SARS-CoV-2-RBD interface [33]. It was shown that mutation of a hydrophobic residue V404 in the SARS-CoV to K417 in SARS-CoV-2 can create a salt bridge across the hydrophobic contact region and leads to a greater electrostatic complementarity and binding affinity of the SARS-CoV-2 complex with human ACE2 enzyme. Microsecond, all-atom MD simulations of the full-length SARS-CoV-2 S glycoprotein embedded in the viral membrane, with a complete glycosylation profile have been recently reported, providing the unprecedented level of atomistic details, showing differential accessibility of the RBD regions to the glycan shield in the open and closed forms [34]. This study demonstrated that the dynamics of glycan shield can be allosterically coupled to conformational changes, regulating the equilibrium transitions and response to the host receptor. MD simulations of the SARS-CoV-2 spike glycoprotein identified the changes in the molecular properties due to conformational flexibility [35]. Several computational methods were used to identify potential allosteric sites on the SARS-CoV-2 spike protein [36] and employed network modeling to suggest allosteric signaling as a possible mechanism underlying virus transmission in the SARS-CoV-2 spike proteins [37]. Although computational studies provided interesting insights into the molecular nature of the binding interactions, a clear consensus and quantitative understanding of the key determinants of the binding affinity and selectivity has not been reached. Nonetheless, these investigations suggested that a balance of the conformational dynamics, protein stability effects, and the relative interaction strength may drive stronger binding of the SARS-CoV-2 RBD with the human ACE2 receptor. While the structural details of the binding interface have been established, the quantitative characterization and classification of these energetic contributions and their potential synergies in determining the binding mechanisms are not fully understood and will be examined in this study.

In this work, we perform a computational investigation of the SARS-CoV-RBD and SARS-CoV-2-RBD binding with human ACE2 receptor using modeling of coevolutionary networks, coarse-grained and all-atom MD simulations, and binding energetics computations based on atomistic simulations of the unbound and bound protein states. We systematically examine the molecular determinants of the binding mechanism between SARS-CoV-2 and ACE2 through the lens of coevolution and conformational dynamics that conspire to drive cooperative binding interactions and signal transmission. Through simulations and systematic mutational scanning of protein residues, we show that the binding interactions and affinity of SARS-CoV-2-RBD with ACE2 may be significantly influenced by cumulative changes from a large number of residues broadly distributed across the binding interface. The results of this study show that despite structural similarities between spike proteins, SARS-CoV-2-RBD can act as plastic and versatile modulator of virus entry in which dynamic interactions with ACE2 can be mediated through a non-trivial interplay of recognition energy hotspots and broad binding interfaces. Our results provide a robust dynamic mapping of binding interfacial changes and allosteric interactions that may aid in rationalization of the binding preferences and high infectivity of SARS-CoV-2.

## 2. Results and Discussion

### 2.1. Sequence Analysis Links Evolutionary Patterns in SARS-CoV Spike Proteins with Shared Conserved Hotspots at the Binding Interface

**Figure 4:**
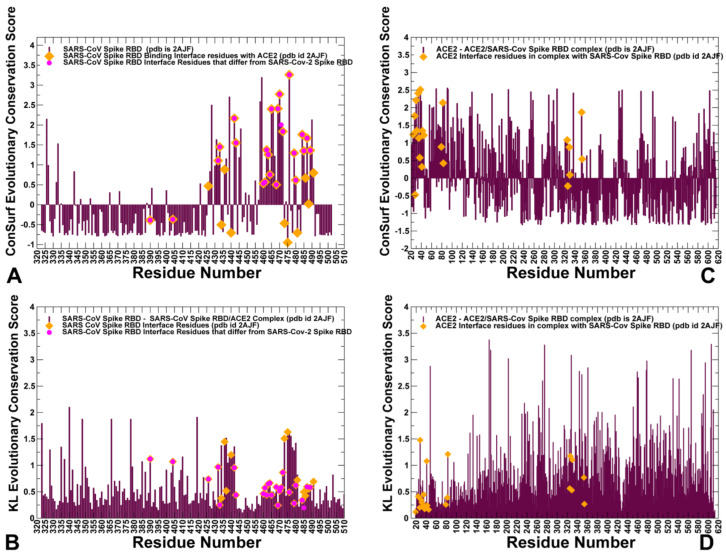
Sequence conservation profiles of the SARS CoV and ACE2 proteins. (**A**) The normalized ConSurf conservation scores for SARS-CoV-RBD spike protein are projected onto the SARS-CoV-RBD complex with ACE2 (residue numbering as in pdb id 2AJF). The ConSurf profiles are shown in maroon bars. The negative ConSurf scores correspond to highly conserved sites (with ConSurf score < 0), and high positive scores (Consurf score > 1.0) depict highly variable positions. Consurf score = 1.0 is defined as a threshold for differentiating moderately and highly variable positions. The binding interface residues with ACE2 are highlighted in orange filled diamonds. The position of the binding interface residues that differ between SARS-CoV-RBD and SARS-CoV-2-RBD are shown in smaller magenta filled circles. (**B**) The KL conservation score for SARS-CoV-RBD spike protein. High KL scores indicate highly conserved sites (above threshold of KL=1.0) and low scores correspond to more variable positions. The annotation of the binding interface residues is as in panel A. (**C**) The normalized ConSurf conservation scores for ACE2 residues (residue numbering corresponds to ACE2 structure in complexes with SARS-CoV (pdb id 2AJF) and SARS-CoV-2 (pdb id 6M0J). The ACE2 binding interface residues are highlighted in orange filled diamonds. (**D**) The KL conservation score for ACE2 residues. The ACE2 binding interface residues are highlighted in orange filled diamonds.

To quantify these observations, we performed sequence alignment analysis and highlighted differences between SARS-CoV and SARS-CoV-2 sequences (Figure 2). ConSurf approach has shown a robust performance in evolutionary analysis of proteins and the latest version of this method [38,39] was used in our study. The evolutionary analysis yielded an overall sequence identity of 78%. The functional domains of the SARS-CoV sequence showed some differences, revealing that the S1 subunit had ≈66% identity, while S2 fusion domain is considerably more conserved with 92% identity. The sequence conservation of S1 RBD yielded ≈73% identity, while the RBM region involved in direct interactions with the ACE2 receptor displayed a more significant variability as similarity between SARS-CoV and SARS-CoV-2 sequences dropped to ≈50% (Figure 2). This analysis is fully consistent with previous studies [40,41,42] suggesting that a significant sequence variability of the SARS-CoV interacting residues from RBM motif may be accompanied by the corresponding local conformational changes. Structural studies confirmed these assertions revealing small but important changes in the RBM residues of SARS-CoV-RBD and SARS-CoV-2-RBD proteins [21,22,23,24,25,26]. We utilized two different sequence conservation measures Consurf score [38,39] and KL sequence conservation score [43,44,45,46,47] to identify regions of high conservation and characterize in some details the extent of conservation and variability of the binding interface residues (Figure 4). We analyzed the results of sequence analysis in the context of structural and functional information, particularly to quantify the conservation patterns of the hotspot residues and uncover evolutionary origins of SARS-CoV-2 binding selectivity with ACE2. Structural studies established that the core of the SARS CoV spike protein is formed by a five-stranded antiparallel β sheet (β1 to β4 and β7), with three short connecting α helices exhibiting significant sequence conservation (Figure 1). The disulfide bonds C323-C348, C366-C419 (in atom numbering of SARS-CoV-RBD, pdb id 2AJF) are formed by fairly conserved cysteine residues (Figure 4A,B). Interestingly, one of the bridges C467-C474 residues in the binding region and is anchored by a two-stranded β sheet (β5 and β6) with these cysteine residues showing a moderate variability (Figure 4A). In particular, we further examined the sequence conservation profile of the RBM region (residues 424–494) that displayed a significant variability. Notably, the RBM region is tyrosine rich (Y436, Y438, Y440, Y442, Y454, Y475, Y481, Y484 in SARS-CoV-RBD). While many of these residues make interactions with ACE2, only two sites Y442 and Y484 are replaced in the SARS-CoV-2-RBD by L455 and Q498, respectively (Figure 3B,C). The sequence analysis confirmed that the majority of RBM tyrosine residues are conserved. However, Y442 and Y484 positions yielded positive ConSurf conservation scores, indicating that these positions are variable and may contribute to the binding affinity differences between SARS-CoV-RBD and SARS-CoV-2-RBD complexes with ACE2 (Figure 4A). We specifically highlighted Consurf and KL conservation score values for the RBD residues, including those positions that differ between SARS-CoV-2 and SARS-CoV proteins (Figure 4A,B). As expected, the substituted interacting residues in the RBM region displayed an appreciable sequence variability. Moreover, the binding interface residues that differ between SARS-CoV-2-RBD and SARS-CoV-RBD form clusters of variable positions (corresponding to high positive Consurf scores and, respectively, low KL scores). Hence, despite structural similarities of the binding interfaces, the individual modifications in the multiple variable positions may lead to a number of moderate interaction changes that could collectively bring about a significant cumulative contribution and contribute to the binding selectivity of the SARS-CoV-2-RBD protein with ACE2 host receptor.

According to our results, sequence analysis using ConSurf and KL evolutionary conservation metrics yielded a consensus by identifying several binding interface residues as conserved hotspots. Indeed, both methods pointed to a group of conserved hotspot residues from the RBM region including K390, V404, Y440, N473, Y475, and Y481 sites in SARS-CoV-RBD (Figure 4A,B, Table 1). The strategically located Y440, N473, Y475, and Y481 hotspot residues anchor the RBM region and are shared between SARS-CoV-RBD and SARS-CoV-2-RBD proteins (Table 1). Some of these residues belong to a conserved segment 472-LNCYWPL-478 in SARS-CoV (486-FNCYFPL-492 in SARS-CoV-2) in which Y475 position (Y489 in SARS-CoV-2) emerged as a central binding energy hotspot (Figure 4A). It is worth noting that segment 472–486 forms a loop that is not present in certain S-proteins of coronavirus isolated in bats [40]. This loop extends the interaction area between SARS-CoV-2-RBD and human ACE2 and can contribute to binding interactions. The conserved segment is flanked by F486 in SARS-CoV-2 which is a conservative replacement of L472 in SARS-CoV and is implicated as an important contributor of binding affinity [14]. The original structural studies of SARS-CoV spike protein in the complex with ACE2 suggested that residues L472, N479, and T487 that are immediately adjacent to the conserved patch could be critical for binding selectivity [14,24,25,26]. These positions in SARS-CoV-2 correspond to F486, Q493, and N501 residues involved in critical interactions of SARS-CoV-2-RBD with ACE2 (Figure 3A,D).

The structure of ACE2 consists of the N-terminus (residues 19–102, 290–397, and 417–430) and C-terminus subdomain II (residues 103–289, 398–416, and 431–615) that form the opposite sides of the active site cleft [48,49,50,51]. The conservation patterns for ACE2 using both metrics revealed a cluster of conserved residues in the subdomain I (Figure 4C,D). The ACE2 interaction sites with SARS-CoV-RBD proteins involve α-helices of ACE2 (residues 20–52, 56–86, 324–331) and β-strand (residues 341–361). We quantified the interface contacts between SARS-CoV-RBD/SARS-CoV-2RBD and ACE2 using PRODIGY approach [52,53] that counts the number of interatomic contacts at the interface of a protein–protein complex within a 5.5 Å distance threshold (Tables S2 and S3). The list of the intermolecular contacts showed that the total number of contacts made by SARS-CoV-2 RBD with ACE is only moderately greater than the ones formed by SARS-CoV-RBD (Tables S2 and S3). Among ACE2 residues involved in the interfacial contacts several interacting residues are relatively conserved (F28, Y41, Y83, G326, N330), while some other interfacial sites (T27, D30, K31, H34, D38) are more variable (Figure 4C,D). The hydrophobic ACE2 residues buried in the central segment of the binding interface tend to be more conserved, where residues F28, L79, Y83 form contacts with Y489, N487, and F486 of SARS-CoV-2 (Figure 3C,D). Another important conserved ACE2 site corresponds to Y41 position that together with Y505 in SARS-CoV-2-RBD “sandwich” the recognition hotspot bridges K353-D38 (Figure 3A). At the same time, some other ACE2 residues at the interface periphery could be more variable and tolerant to conserved amino acid substitutions. These results are consistent with deep mutagenesis studies, where a number of ACE2 variants with the increased binding to the RBD were identified, suggesting that sequence of human ACE2 is only suboptimal for binding of SARS-CoV spike proteins [28]. This experimental study discovered feasibility of hydrophobic substitutions at T27 position, potential of D30E modification, and tolerance to aromatic substitutions at K31 position. Consistent with these revelations, sequence analysis showed that these ACE residues featured positive Consurf scores for T27, D30, and K31 pointing to their evolutionary variability (Figure 4C,D).

To summarize, the results indicated that several conserved sites could form important recognition hotspots of the SARS-CoV binding interface with ACE2. We also proposed that binding free energy differences between SARS-CoV and SARS-CoV-2 RBD may be influenced by cumulative contributions of large number of variable positions. Although sequence analysis can identify important variable RBM regions implicated in binding, the extent of sequence variability may not necessarily directly correlate with the functional importance of respective residues for binding selectivity. In general, sequence analysis demonstrated that the extensive binding interface between SARS-CoV-RBD and ACE2 proteins can involve contributions of both conserved and more variable positions that are broadly distributed and collectively drive the binding mechanism in which sequence variability and structural plasticity can be important for rendering binding selectivity of SARS-CoV-2-RBD protein.

### 2.2. Coevolution and Mutual Information Interdependences of the SARS-CoV-RBD and ACE2 Suggest Potential Regulator and Transmitter Sites of Binding and Communication

**Figure 5:**
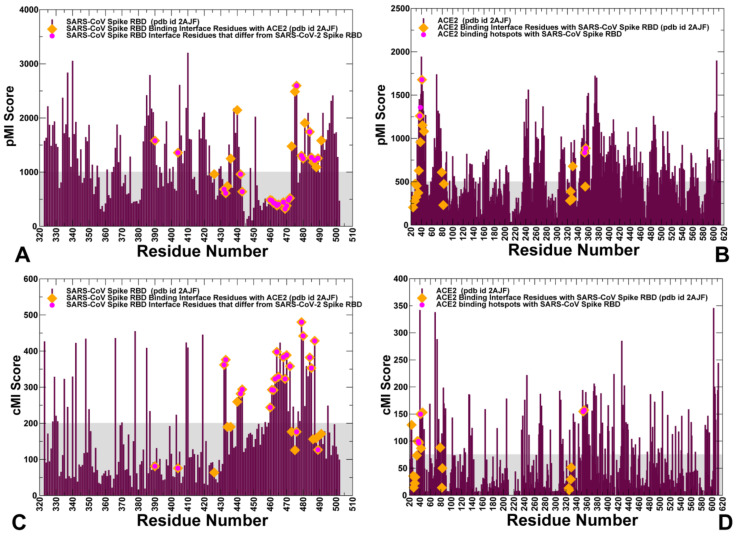
Coevolutionary profiles of the SARS CoV and ACE2 proteins. (**A**) The structure-based pMI profile for SARS-CoV-RBD spike protein is projected onto the SARS-CoV-RBD complex with ACE2 (residue numbering as in pdb id 2AJF). The binding interface residues that make contacts with ACE2 are highlighted in orange filled diamonds. The position of the binding interface residues that differ between SARS-CoV-RBD and SARS-CoV-2-RBD are shown in smaller magenta filled circles. (**B**) The pMI profile for ACE2 residues (pdb id 2AJF). The annotation of the binding interface residues is as in panel A. (**C**) The sequence-based cMI profile for SARS-CoV-RBD is projected onto the SARS-CoV-RBD complex with ACE2 (pdb id 2AJF). (**D**) The cMI profile for ACE2 residues (residue numbering corresponds to ACE2 structure in the complex with SARS-CoV (pdb id 2AJF). The annotation of the binding interface residues is as in panel A. The horizontal lines on the graphs are materialized in cyan to indicate thresholds used to differentiate moderate and high values of pMI and cMI scores.

The important complementary information can be gained by considering coevolutionary dependencies of protein residues in which functional and structural changes are often accompanied by compensatory mutations at the protein–protein interfaces to modulate activity. Coevolving residues can mediate protein recognition in multiprotein complexes and are often spatially close to each other, forming clusters of interacting residues that are located near functionally important sites [54,55,56,57]. The coevolving residues may be also clustered in mobile regions surrounding structurally stable functional sites and form interaction networks of evolutionarily coupled residues that facilitate protein conformational changes [58]. Using coevolutionary residue matrices determined by the shared mutual information, we constructed the network of coevolutionary couplings in the SARS-CoV-RBD complexes in which the nodes represented protein residues and links corresponded to coevolutionary dependencies (or extent of shared mutual information) between these residues [59]. For each residue, we computed cumulative mutual information (cMI) parameter, that evaluates the shared mutual information between a given residue and other protein residues, and proximity-based mutual information parameter (pMI) approximating the amount of mutual information shared by a given residue with the structurally close neighboring nodes [43,44]. Using this approach, we characterized mutual information interdependencies between functionally important regulatory sites and identified clusters of residues involved in coevolutionary couplings. The computed sequence-based cMI and structure-based pMI parameters (Figure 5) provided several important insights that expanded our understanding of evolutionary traits at the binding interface regions. According to our results, the high pMI interfacial residues are largely conserved and are aligned with the central segment of the interface for SARS-CoV-RBD (Y436, Y440, Y475, W476, Y481, Y484) and SARS-CoV-2-RBD (Y449, Y451, Y489, F490, Y495, Q498) (Figure 5A,C). It is also evident that high pMI sites are enriched by conserved tyrosine residues located at the intermolecular interface. Owing to their conservation, these high pMI positions are located at the epicenter of coevolutionary couplings and are characterized by local structural environment that is enriched by coevolving residues (Figure 5A,B). According to our previous studies [59,60,61] high pMI positions are often aligned with the structurally stable regulatory centers that dictate which channels of signal transmission are activated in coevolutionary networks. The results of the current analysis suggested that these conserved interface residues may be required for recognition during virus entry into the host receptor and structural stabilization of the binding interface around these key anchor points. Structural positions of these sites in the central segment of the binding interface may provide the necessary “stabilization anchors” that hold SARS-CoV-RBD and SARS-CoV-2 RBD proteins in the recognition mode. We observed that a number of more variable binding residues in the vicinity of the “anchor” sites displayed high cMI values, indicating that the interfacial regions structurally proximal to more conserved central residues may leverage coevolutionary dependencies to facilitate binding with ACE2 (Figure 5C). Many residues from the recognition loop of the RBM (residues 470–491 in SARS-CoV-2) featured high cMI values (Figure 5B, Table 2) showing that this flexible region is enriched by sites sharing mutual information and therefore may be critical for signal transmission and binding. Among high cMI residues in the SARS-CoV-RBD are L472, N479, and T487 (F486, Q493, and N501, respectively, in SARS-CoV-2-RBD) (Figure 5C, Table 2). These RBM residues showed appreciable sequence variability and were also aligned with the cMI peaks, (Figure 5C, Table 2). Furthermore, these coevolving RBM residues form a network of interactions with Y41, K353, E37, D38, E35, and K31 residues that also displayed high cMI values in ACE2 (Figure 5D).

**Figure 6:**
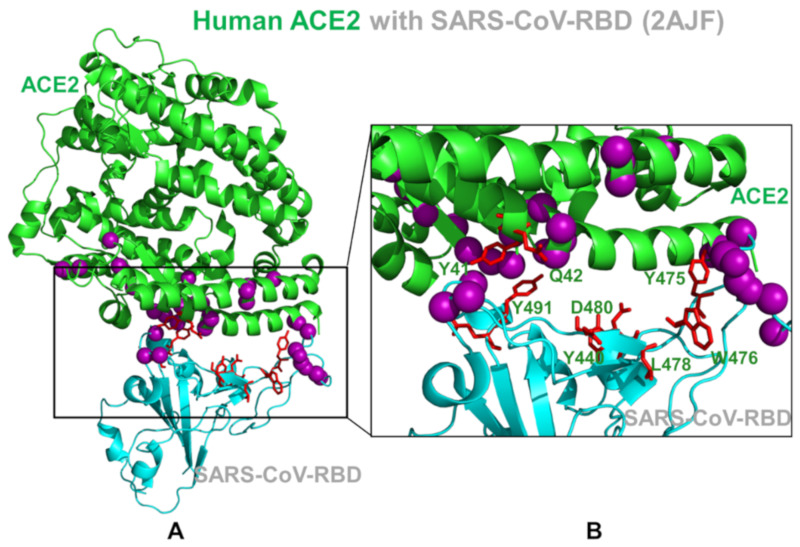
Structural mapping of the SARS-CoV-RBD and ACE2 residues with high pMI and cMI values. (**A**) A general overview of the SARS-CoV-RBD complex with ACE2. The high pMI residues serving as regulators of coevolutionary network are shown in pink spheres. The highly coevolving residues with high cMI values are shown in red sticks. Note a consolidation of regulatory high pMI sites in the central segment of the binding interface and clusters of highly coevolving residues near the flexible loop regions of the binding interface. (**B**) A close-up of the binding interface between SARS-CoV-RBD and ACE2. The high pMI residues (pink spheres) and high cMI residues (red sticks) are shown and annotated for the key positions that control coevolutionary couplings in the complex.

Our findings support the assertion that these clusters of coevolving mobile residues are important for binding and could be implicated in the transmission of virus signals. By mapping residues featuring high pMI and cMI values, we observed that structurally stable and conserved residues Y475, W476, L478 in SARS-CoV (Y489, F490, and L492 in SARS-CoV-2) are located in the middle of the binding interface and could form a cluster of high pMI sites (Figure 6). The proximity-based coevolutionary signature of these sites occupying strategic interface positions may be associated with their functional role as regulators of signaling between interacting proteins in the coevolutionary network.

This specific signature of functional sites could make them indispensable for the integrity of coevolutionary interaction networks, which may cause even minor mutations at these positions to be highly detrimental. We argue that mutations in these conserved tyrosine positions may interfere with both the stability and recognition of the virus protein and drastically change binding for both SARS-CoV spike proteins. We observed that highly coevolving residues in SARS-CoV-RBD tend to occupy the mobile loops of the binding interface and may be involved in the conformational and interaction rearrangements during binding with ACE2 (Figure 6). These residues are only moderately conserved and could act as flexible carriers of allosteric signals between interacting proteins. Structural mapping highlighted a dense cluster of RBD residues from the mobile interacting loop that is enriched by coevolving residues in SARS-CoV-2-RBD (Figure 6). A cluster of highly coevolving centers can be also seen in the β-hairpin region of ACE2 (residues 348–360) as residues R357, D355, and K353 feature high cMI values and may be involved in the transmission of the binding signal. Based on our observations, this cluster of highly coevolving interacting residues in the β-hairpin region of ACE2 may be involved in propagation of functional signals across the binding interface and serve as a mediating cluster for virus transmission. In this model, the flexibility of highly coevolving residues sharing a significant degree of mutual information may allow for compensatory mutations in the interfacial regions to secure structural features that control allosteric signaling between interacting proteins in the complex. We argue that the observed interplay of evolutionary and coevolutionary signatures for the binding interface residues may contribute to their distinct functions as regulators and transmitters of allosteric signaling and govern binding selectivity of the SARS-CoV-2 protein.

### 2.3. Conformational Dynamics of the SARS-CoV and SARS-CoV-2 RBD Proteins: A Comparative Analysis of ACE2-Induced Protein Mobility and Dynamics-Driven Allostery

**Figure 7:**
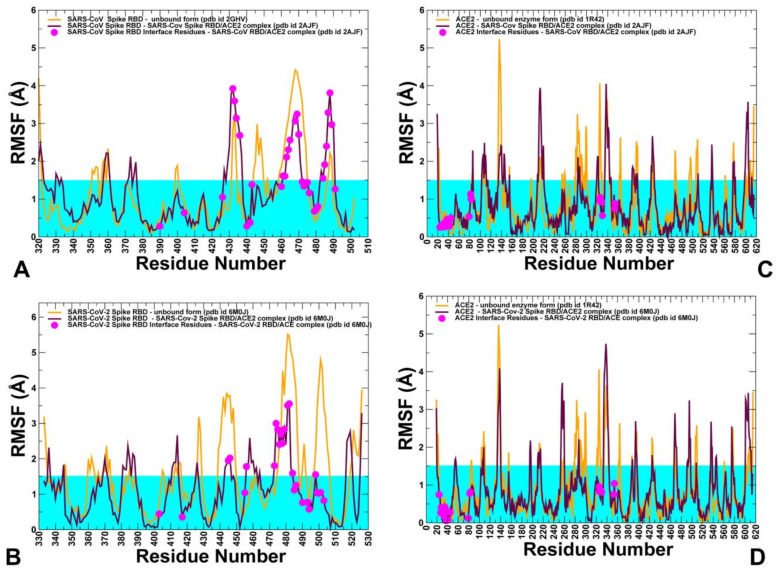
All-atom conformational dynamics of the unbound and bound SARS-CoV structures. The root mean square fluctuations (RMSF) profiles are obtained from all-atom MD simulations of the crystal structures. (**A**) Conformational dynamics profiles of the unbound SARS-CoV (pdb id 2GHV) (in orange lines) and SARS-CoV-RBD in the complex with ACE2 (pdb id 2AJF) (in maroon lines). The positions of the SARS binding interface residues are highlighted in magenta filled circles. (**B**) The RMSF dynamics profiles of the unbound SARS-CoV-2 (in orange lines) and SARS-CoV-2 RBD in the complex with ACE2 (pdb id 6M0J) (in maroon lines). The positions of the SARS binding interface residues are highlighted in magenta filled circles. (**C**) The RMSF dynamics profiles of the unbound ACE2 (pdb id 1R42) (in orange lines) and in the complexes with SARS-CoV-RBD (in maroon lines). The ACE2 binding interface residues are shown in magenta filled circles. (**D**) The RMSF dynamics profiles of the unbound ACE2 (pdb id 1R42) (in orange lines) and in the complexes with SARS-CoV-2 RBD (in maroon lines). The ACE2 binding interface residues are shown in magenta filled circles. The horizontal lines on all graph panels are materialized in cyan to indicate thresholds used to differentiate small and higher thermal motions.

**Figure 8:**
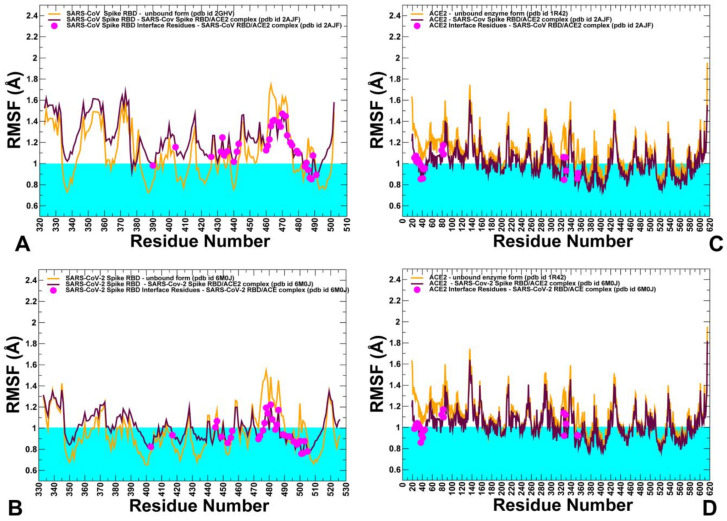
CG conformational dynamics of the unbound and bound SARS-CoV structures. The RMSF profiles are obtained from CG simulations of the crystal structures. (**A**) Conformational dynamics profiles of the unbound SARS-CoV (pdb id 2GHV) (in orange lines) and SARS-CoV-RBD in the complex with ACE2 (pdb id 2AJF) (in maroon lines). The positions of the SARS binding interface residues are highlighted in magenta filled circles. (**B**) The RMSF dynamics profiles of the unbound SARS-CoV-2 (in orange lines) and SARS-CoV-2 RBD in the complex with ACE2 (pdb id 6M0J) (in maroon lines). The positions of the SARS binding interface residues are highlighted in magenta filled circles. (**C**) The RMSF dynamics profiles of the unbound ACE2 (pdb id 1R42) (in orange lines) and in the complexes with SARS-CoV-RBD (in maroon lines). The ACE2 binding interface residues are shown in magenta filled circles. (**D**) The RMSF dynamics profiles of the unbound ACE2 (pdb id 1R42) (in orange lines) and in the complexes with SARS-CoV-2 RBD (in maroon lines). The ACE2 binding interface residues are shown in magenta filled circles. The horizontal lines on all graph panels are materialized in cyan to indicate thresholds used to differentiate small and higher thermal motions.

To explore conformational landscapes of the SARS-CoV interactions with human ACE2 in sufficient details, we performed both atomistic MD simulations (Figure 7) and coarse-grained (CG) simulations (Figure 8) of the SARS-CoV and SARS-CoV-2 structures in their unbound and bound forms. All-atom MD simulations of the SARS-CoV structures in different functional states can provide a rigorous quantitative assessment of atomistic fluctuations and interaction details. However, these simulations can become time-consuming when performed for multiple SARS-CoV structures and complexes under various simulation conditions and environments. To complement all-atom MD simulations and compare quantitative and qualitative insights gained from molecular simulations, we also performed multiple CG simulations of the SARS-CoV and SARS-CoV-2 structures and their complexes with ACE2 (Figure 8). Among objectives of this analysis was to analyze the extent to which CG simulations can capture and reproduce both quantitative and qualitative patterns in the atomistic dynamics and therefore represent a powerful complementary alternative for molecular studies of the SARS-CoV-2 binding and interactions. 

Using these simulation approaches applied to the structures of SARS-CoV and SARS-CoV-2 RBD proteins in the unbound and ACE2-bound forms, we performed a detailed comparative analysis of the conformational dynamics profiles and examined salient features of the dynamic conformational landscapes. In MD simulations of the SARS-CoV-RBD with ACE2, the structure of the complex fluctuated within fairly narrow RMSD values until it reached a plateau after about 300 ns and continued to maintain this equilibrium through the remaining simulation time (Figure S1A). The structural integrity and thermal stability of the SARS-CoV-2-RBD complex with ACE2 during MD simulations was retained with RMSD < 4.5 Å from the starting crystal structure (Figure S1B). The simulations reached the first plateau with RMSD ≈3.5 Å after ≈300 ns followed by the increased fluctuations at ≈600 ns and subsequent thermal stabilization with RMSD ≈4.5 Å from the crystal structure (Figure S1B). Atomistic simulations revealed a dynamic nature of the SARS-CoV/SARS-CoV-2 structures that maintained a considerable degree of residual mobility (Figure 7). CG simulations displayed generally more stable profiles of the SARS spike protein and ACE2 in their unbound forms, pointing to the increased stabilization of the binding interface residues in the complexes (Figure 8). The conserved core of SARS-CoV-RBD consists of five antiparallel β strands (β1 to β4 and β7), with three connecting α-helices (Figure 1). Atomistic MD simulations showed that these regions (residues 341–344, 364–367, 383–390, 493–500, 394–403, 431–438 in SARS-CoV-RBD) are stable and structurally virtually indistinguishable between the SARS-CoV and SARS-CoV-2 proteins (Figure 7A,B).

Nonetheless, both all-atom simulations and CG simulations (Figure 8A,B) revealed some moderate thermal motions in the core of SARS-CoV-RBD and displayed greater structural rigidity for core regions in the SARS-CoV-2-RBD complex with ACE2. The antiparallel β-sheets (β5 and β6) (residues 439–441 and 478–481 in SARS-CoV-RBD and residues 451–454 and 492–495 in SARS-CoV-2-RBD) that anchor the RBM region to the central core showed a particularly significant stabilization, that was especially noticeable in all-atom MD simulations (Figure 7A,B). By anchoring the RBM region, these strands ensure proper complementarity of the concave surface with the ACE2 enzyme. The thermal stability of these regions is preserved in both the unbound spike forms and complexes with ACE2 enzyme, suggesting that the structural integrity of these antiparallel β-sheets is a prerequisite for establishing functional interfaces with host receptor. All-atom MD simulations also indicated that stability of the protein core in the unbound and bound forms of SARS spike proteins can be comparable. Moreover, smaller fluctuations were seen for the unbound structure in the loop regions that surround the protein core and are not involved in ACE2 interactions (residues 345–382 in SARS-CoV) (Figure 7A,B).

The CG simulation profiles showed that the protein core of five antiparallel β strands (β1 to β4 and β7) can be comparable and even more stable in the unbound SARS-CoV form while the RBM region (residues 423–494 in SARS-CoV and residues 435–506 in SARS-CoV-2) is more flexible (Figure 8A,B). Both atomistic and CG simulations indicated that this region is flexible in the unbound forms of SARS spike proteins, but undergoes stabilization in the ACE2-bound complexes. However, the pattern of the increased stabilization in the RBM region is more revealing in all-atom MD simulations that unambiguously pointed to the greater rigidity of this region in the SARS-CoV-2 RBD complex with ACE2 (Figure 7A,B). Atomistic simulations also revealed important subtle differences in the mobility of RBM residues that are not immediately apparent from CG simulations, indicating that atomistic resolution of thermal fluctuations and interaction contacts may be critical for quantitative understanding of the dynamic differences between structurally similar SARS-CoV-RBD and SARS-CoV-2 RBD proteins. In the SARS-CoV complex with ACE2, several regions from the RBM (residues 425–440, 480–494) displayed fluctuations that were similar in the unbound and bound protein forms, indicating that the interactions in this region with ACE2 are moderate and could tolerate fluctuations in this region (Figure 7A). In the complex, binding contacts may only partly reduce flexibility of SARS-CoV at the interfacial loop 455–476 that still retains a considerable degree of residual mobility (Figure 7A). Interestingly, the conformation of this segment in the SARS-CoV complex is identical to the one adopted in the unbound SARS-CoV form. Hence, the RBM regions in the SARS-CoV complex with ACE2 may be characterized by only a partial reduction of mobility at the binding interface (Figure 7A). The RBM loops in SARS-CoV-RBD (residues 461–471, 474–476, and 480–491) experienced appreciable fluctuations in the complex with ACE2, particularly loop 480–491 displaying higher fluctuation with respect to the rest of the RBD structure. These RBM regions in the SARS-CoV-2 RBD protein (residues 474–485, 488–490, and 494–505) featured noticeably smaller fluctuations, especially for the ridge loop 494–505 that becomes largely immobilized upon gaining productive stabilizing contacts with the host receptor ACE2 (Figure 7B). A generally similar trend could be also inferred from CG simulations where the RBM interface residues in the SARS-CoV-RBD experienced thermal fluctuations above the rigidity threshold of 1.0 Å (Figure 8A) while the corresponding positions in the SARS-CoV-2-RBD complex become immobilized with RMSF fluctuations of 0.7–0.8 Å (Figure 8B).

**Figure 9:**
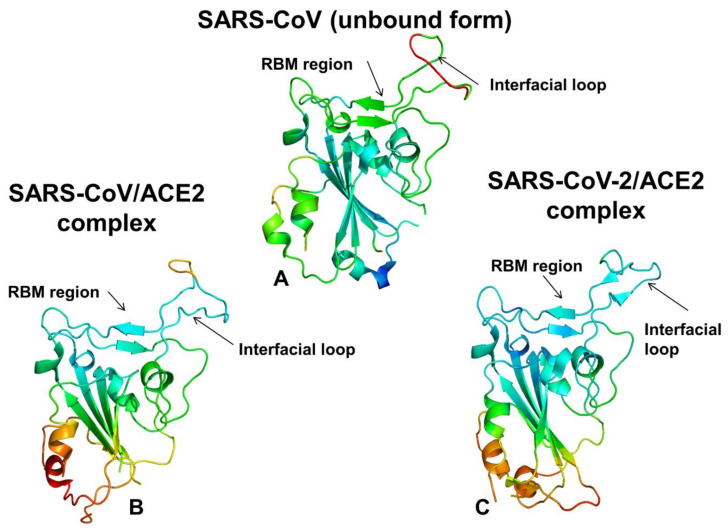
Structural mapping of the conformational mobility profiles in the SARS-CoV unbound structure and SARS-CoV-RBD/SARS-CoV-2 RBD complexes with ACE2 obtained from all-atom MD simulations. (**A**) Conformational dynamics profiles mapped on the unbound form of SARS-CoV-RBD (pdb id 2GHV). A ribbon-based protein representation is used with coloring (blue-to-red) according to the protein residue motilities (from more rigid-blue regions to more flexible-red regions). (**B**) Structural map of the conformational mobility profile of SARS-CoV-RBD in the complex with ACE2. The more stable regions are shown in blue and more flexible are in green-to-red coloring scale. (**C**) Structural map of dynamics profile of SARS-CoV-2-RBD in the complex with ACE2. The more stable regions are shown in blue and more flexible are in green-to-red coloring scale. For each panel, the position of the RBM region and the interfacial loop, which is an element of the RBM, are indicated with respective arrows. Note the redistribution of conformational mobility between the unbound and bound forms for SARS-CoV and SARS-CoV-2.

Importantly, both coarse-grained and atomistic simulations consistently revealed a stronger stabilization of the RBM residues for SARS-CoV-2 spike protein in the complex with ACE2 (Figure 7B and Figure 8B). In this case, the interfacial loop segments (residues 436–455 containing an important motif 444-KVGGNYNY-451 and residues 470–506 that feature the interacting motif 495-YGFQPTNG-502 in SARS-CoV-2-RBD) displayed significantly reduced fluctuations as compared to SARS-CoV Spike RBD (Figure 7B). As a result of the host receptor-induced stabilization, a significant number of SARS-CoV-2 residues across the entire interface (G446, Y449, F486, Y489, G496, Q498, T500, N501, and G502) become rigidified and are positioned to make strong specific interactions with ACE2. In addition, a portion of the interfacial loop (residues 473–487) that is mobile in the unbound form undergoes a significant stabilization and adopts a different conformation in the SARS-CoV-2-RBD as compared to the SARS-CoV-RBD complex (Figure 9). These changes position SARS-CoV-2 residues Y473, A475, G476 to establish contacts with ACE2. Moreover, SARS-CoV-2 residues Y489 and F486 pack against F28, L79, M82, and Y83 on ACE2 to form a critical patch of hydrophobic interactions at the interface (Figure 3D). Importantly, not only these residues showed markedly reduced fluctuations, but the large interfacial stretch of residues across entire binding interface (residues 486-FNCYFPLQSYGFQ-498) including key Q493 and Q498 interacting sites exhibited a stronger stabilization as compared to their respective counterparts in SARS-CoV (Figure 7A,B). Indeed, the corresponding residue stretch 472-LNCYWPLNDYGFY-484 in the SARS-CoV displayed a greater mobility and is marked by numerous amino acid changes between SARS-CoV-2 and SARS-CoV (F486 vs. L472, F490 vs. W476, Q493 vs. N479, S494 vs. D480, Q498 vs. Y484, respectively). Hence, atomistic simulations established that significant structural similarity between SARS-CoV and SARS-CoV-2 bound complexes with ACE2 may be accompanied by subtle and yet noticeable dynamic changes that may be important for driving binding selectivity preferences of SARS-CoV-2 RBD protein. Hence, atomistic and CG simulations indicated that the RBM residues in the SARS-CoV-2 RBD protein undergo a more significant structural stabilization upon ACE2 binding as evident by marked differences in the fluctuations of the unbound and bound spike protein. In some contrast, the corresponding dynamic changes between the unbound and bound forms of the SARS-CoV-RBD are markedly smaller, suggesting that a considerable degree of protein mobility can be retained in the complex, including some variability at the binding interface.

Conformational dynamics profiles of ACE2 residues were very similar in the unbound and bound forms as evident from both atomistic simulations (Figure 7C,D) and CG simulations (Figure 8C,D). The protein core residues displayed significant stability and only small changes were observed for the binding interface residues in ACE2. These segments of ACE2 were stable in both unbound and bound forms (Figure 7C,D). The two ridges of the RBM interact with the ACE2 receptor by contacting the inter-helical loops on one side and a unique β-hairpin (residues 348–360) on the other side of the interface. The small portion of the β-hairpin turn containing hotspot residue K353 may be relatively flexible in the unbound ACE2 form when structural restraints from viruses are absent. Among important observations revealed by both atomistic and CG simulations is a noticeable redistribution of conformational dynamics in the SARS-CoV and SARS-CoV-2 structures upon complex formation with ACE2. The exchange of conformational mobility is manifested by stabilization of the ACE-2 bound RBM regions and the increase of thermal motions in the rest of the protein. Moreover, such redistribution of thermal fluctuations is more pronounced in SARS-CoV-2-RBD that is coupled with a stronger stabilization of the entire binding interface. These findings imply that binding of SARS-CoV-2 spike with the host receptor may exhibit signs of a dynamically driven allosteric mechanism which is typically exemplified by the lack of structural changes between the unbound and bound forms, coupled with an exchange of conformational mobility between local protein regions [62,63,64,65]. This hypothesis requires more extensive work and detailed analysis of the collective dynamics and binding-induced changes in the organization and modularity of dynamic interaction networks for the unbound SARS-CoV-RBD and ACE proteins and complexes. This extends beyond the scope of the present study and will be presented elsewhere. In this context, our most recent study of the SARS-CoV-2 spike trimers using perturbation analysis and network modeling presented evidence that the SARS-CoV-2 spike protein can function as an allosteric regulatory engine that fluctuates between dynamically distinct functional states [37].

To determine dynamic patterns of the key binding interface contacts, we monitored the interfacial residue contact stability during all-atom MD simulations by computing the productive contact time throughout the MD trajectory of the SARS-CoV-RBD complex with ACE2 (Table S4) and SARS-CoV-2 RBD complex (Table S5). This analysis revealed the larger number of persistent stabilizing contacts in the SARS-CoV-2 RBD complex with ACE2, where most of the interfacial interactions remained stable throughout the entire simulation period (Table S5). In some contrast, some interfacial contacts in the SARS-CoV-RBD complex could form, break, and then reestablish during the simulation period (Table S4). Of particular interest is dynamics of contacts for binding hotspots that involve K31 and K353 residues of ACE2. In the ACE2 enzyme, K31 forms the intramolecular salt bridge with E35 while K353 forms a salt bridge with D38. These intramolecular bridges are preserved in the complex with SARS-CoV-RBD. In the SARS-CoV complex with ACE2, K31 hotspot in ACE2 maintains stable interactions with Y442 and Y475 of SARS-CoV-RBD (Table S4). Strikingly, the corresponding positions in the SARS-CoV-2 RBD (L455 and Y489, respectively) are unable to establish persistent stable contacts with K31 even though simulations registered a close structural overlap for corresponding sites in the SARS-CoV-2 and SARS-CoV-RBD. The loss of the intramolecular salt bridge K31-E35 broken in the structure of the SARS-CoV-2 complex with ACE2 is counterbalanced by new contacts made by E484, Q493, F456, and F490. Several of these contacts are more persistent in their duration (E484-K31 and Q493-K31), while interaction contacts with hydrophobic residues F456 and F490 are more dynamic (Table S4). Indeed, the interaction between Q493 of SARS-CoV-2 and K31 hotspot of ACE2 was reported in the experimental studies [15]. These results are consistent with recent MD simulations of the SARS-CoV-RBD complexes [66].

We also report the residue solvent-accessible surface (SASA) in the SARS-CoV-RBD and SARS-CoV-2 RBD complexes that were obtained by averaging the SASA computations over the length of MD simulation trajectories (Figure S2). This analysis helped to evaluate the average buriedness of the interfacial residues in the course of simulations. Among deeply buried residues with small SASA in the SARS-CoV complex are binding interface positions Y440, Y442, L443, F460, Y481, Y484 residues (Figure S2A). At the same time, a number of residues in this region can be partially exposed. Consistent with dynamics profiles, the antiparallel β-sheets β5 and β6 (residues 439–441 and 478–481) in the central segment of the interface showed relatively small SASA ≈15–20 Å^2^, although K439 and R441 featured larger SASA values of 69.7 and 61.9 Å^2^, respectively (Figure S2A). In the central region of the interface residues V404, Y442, F460 are deeply buried, while several other positions (W476 and N479) showed appreciable solvent accessibility. These antiparallel β-sheet regions in the SARS-CoV-2 (residues 451–454 and 492–495) are well buried at the interface with SASA values ≈5–10 Å^2^ (Figure S2C). The RBM residues in the central segment (K417, L456, F456, Y473, F490, and Q493) showed markedly smaller SASA values than their counterparts in SARS-CoV, particularly for F456 and Q493 that are completely buried (Figure S2A,C). These observations reinforced conformational dynamics analysis, suggesting that the entire binding interface is considerably more rigidified in the SARS-CoV-2 complex owing to structural restrictions to maintain multiple favorable contacts with ACE2. Of particular notice is a comparison of residues L472, N479, and T487 (SARS-CoV) with corresponding positions F486, Q493, and N501 residues in SARS-CoV-2 that showed dynamic differences and are implicated in critical interactions that determine binding preferences of the SARS-CoV-2 complex. In the SARS-CoV complex, N479 residue is fairly exposed with large SASA value of 90.6 Å^2^, whereas the respective site Q493 in SARS-CoV-2 is completely buried with SASA of 0.5 Å^2^. Other residues in this region have similar SASA values, being either completely buried (T487 → N501) or showing an appreciable and similar solvent accessibility of ≈70 Å^2^ and dynamic mobility (L472 → F486). Although this analysis indicated the importance of N479 → Q493 modification and a unique role of Q493 as a strong driver of binding selectivity, we also noticed a broad range of dynamic and solvent exposure variations among RBM residues. These results provided an additional support to our hypothesis that binding affinity of SARS-CoV-2 with ACE2 may arise from a cumulative contribution of many interfacial residues with different dynamic and solvent exposure profiles.

In summary, the central finding of the conformational dynamics analysis is the evidence of a much broader and consistent stabilization across the entire RBM interface for the SARS-CoV-2-RBD protein. According to these results, subtle but important differences in mobility of the binding interfaces for SARS-CoV-2 may not be confined to several spatially localized binding hotspots, but instead appeared to be more broadly distributed across many RBM residues. This may be contrasted with SARS-CoV-RBD dynamics patterns in which ACE2 binding may induce thermal stability and strong interaction contacts in the specific hotspots, while other interface patches could retain considerable mobility and exhibit weaker intermolecular contacts. Based on these observations, we suggested that the stronger binding affinity and the associated virus transmission may arise from cumulative dynamic and energetic changes of many SARS-CoV-2 residues at the binding interface with ACE2 enzyme.

### 2.4. Mutational Scanning and Energetic Analysis of the Binding Interfaces: A Cooperative Effect of Multiple Residues Drives SARS-CoV-2 Binding with Host Receptor

**Figure 10:**
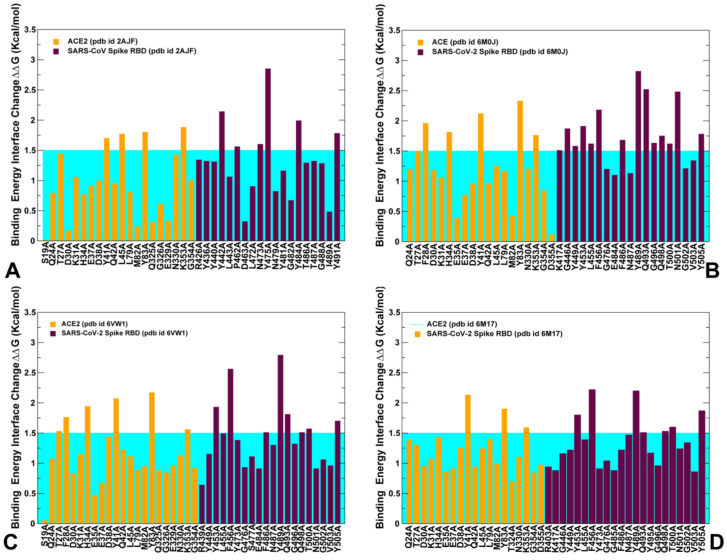
Alanine scanning of the key binding interface residues in the SARS-CoV and SARS-CoV-2 complexes with ACE2. (**A**) The binding free energy changes upon alanine mutations for the interface residues in the SARS-CoV-RBD complex with ACE2 (pdb id 2AJF). The binding energy changes for the ACE residues are shown in orange bars and for the SARS-CoV-RBD interacting residues in maroon bars. The computed values were obtained using BeAtMuSiC approach and were averaged over equilibrium samples from MD simulation. (**B**) The binding free energy changes upon alanine mutations for the interface residues in the SARS-CoV-2 RBD complex with ACE2 (pdb id 6M0J). The binding energy changes for the ACE residues are shown in orange bars and for the SARS-CoV-2 RBD interacting residues in maroon bars. (**C**) The binding free energy changes upon alanine mutations for the interface residues in the engineered chimera SARS-CoV-2 RBD bound with ACE2 (pdb id 6VW1). The binding energy changes for the ACE residues are shown in orange bars and for the SARS-CoV-2 RBD interacting residues in maroon bars. (**D**) The binding free energy changes upon alanine mutations for the interface residues in the SARS-CoV-2 RBD complex with ACE2 obtained from the cryo–electron microscopy structure of the full-length human ACE2 with SARS-CoV-2 RBD in the presence of the neutral amino acid transporter B^0^AT1 (pdb id 6M17). The binding energy changes for the ACE residues are shown in orange bars and for the SARS-CoV-2 RBD interacting residues in maroon bars. The binding free energy changes for each complex are based on the average binding energy estimates over MD trajectories. The error bars for the binding free energy changes were in a small range of 0.05–0.15 kcal/mol. The horizontal lines on the graphs are materialized in cyan to indicate a threshold of 1.5 kcal/mol used to differentiate moderate and large free energy changes and identify key binding energy hotspots.

Based on the detailed analysis of the conformational dynamics, we conjectured that the energetic characterization of the binding interface residues using ensemble-averaged estimates could better quantify binding differences between SARS-CoV-RBD and SARS-CoV-2 RBD. We employed the equilibrium ensembles generated from all-atom MD simulations of the SARS-CoV and SARS-CoV-2 complexes with ACE2 to perform a systematic alanine scanning of the protein residues in the complexes (Figure 10). In particular, we examined the binding free energy changes of the interface residues to determine the hotspot residues and identify differences in the binding interactions between SARS-CoV and SARS-CoV-2 spike proteins. Sequence conservation analysis and conformational dynamics pointed to potentially critical residues at the binding interface that are different between SARS-CoV and SARS-CoV-2 (Figure 3, Table S1). Strikingly, these residues are stretched across the entire interface and include the following important modifications between SARS-CoV and SARS-CoV-2 at the N terminus (R426 → N439, Y484 → Q498, T487 → N501), in the central bridge of the interface (V404 → K417, Y442 → L455, L443 → F456, F460 → Y473, W476 → F490, and N479 → Q493), and in the C terminus (L472 → F486) (Figure 2). While previous studies have highlighted the importance of these positions, the mechanistic role and relative contributions of these residues to protein stability and binding selectivity remain debatable and require further investigation. Additionally, there are several strategically located conserved positions in the middle of the binding interface of SARS-CoV and SARS-CoV-2 (Y440 → Y453 and Y475 → Y489) and N-terminus segment (Y491 → Y505) that are likely to contribute to both protein stabilization and binding affinity of spike proteins.

To aid in a comparative analysis of the binding free energy changes, we performed alanine scanning using dynamic ensembles of multiple SARS-CoV-2 structures bound with ACE2 (Figure 10), including the crystal structure of SARS-CoV-2 spike RBD bound with ACE2 (pdb id 6M0J) [24], the crystal structure of SARS-CoV-2 chimeric RBD bound with human ACE2 (pdb id 6VW1) [25], and the structure of ACE2 with B^0^AT1 and RBD of SARS-CoV-2 (pdb id 6M17) [26]. First, we found that the strongest binding energy hotspots are conserved between SARS-CoV and SARS-CoV-2 proteins and are located in the central segment of the interface (Y440 → Y453 and Y475 → Y489) and N-terminus segment (Y491 → Y505) (Figure 10). These residues are virtually superimposable in the crystal structures and form a very similar number of favorable hydrophobic contacts with ACE2 (Figure 3A,C). Indeed, a large and comparable loss of the binding energy was found for these residues in both SARS-CoV and SARS-CoV-2 (2.82/2.85 kcal/mol for Y475A/Y489A; 1.31/1.91 kcal/mol for Y440A/Y453A; 1.78/1.78 kcal/mol for Y491A/Y505A, respectively) (Figure 10A,B). Importantly, these conserved binding energy hotspots also correspond to the regulatory sites in coevolutionary residue networks that may control signal propagation across binding interfaces. Other important binding residues in the SARS-CoV RBD showing a significant energy loss upon alanine modifications included Y442, N473, and Y484 positions (Figure 10A). In these positions, Y442A change of 2.15 kcal/mol can be compared with a smaller loss for L455A of 1.62 kcal/mol, while L472A with 0.9 kcal/mol change can be contrasted to a larger loss for F486A of 1.68 kcal/mol (Figure 10A,B). Interestingly, in several other important positions, binding free energy losses caused by alanine mutation can decrease in going from the SARS-CoV-RBD to SARS-CoV-2 RBD. For instance, the energy change upon N473A mutation of 1.6 kcal/mol can be opposed to the N487A loss of 1.13 kcal/mol, and Y484A mutational change of 1.99 kcal/mol can be compared to Q498A energy loss of 1.75 kcal/mol (Figure 10A,B). The computed binding energy changes for the SARS-CoV-2 RBD structures consistently predicted residues Q493 and N501 as the strongest energetic hotspots (Figure 10B–D). The important difference in the binding energies resulted from amino acid modification of N479 in SARS-CoV to Q493 in SARS-CoV-2. The binding free energy loss of 2.52 kcal/mol for Q493A mutation can be contrasted to a much smaller change of 0.82 kcal/mol for N479A (Figure 10A,B). The error bars for the binding free energy changes were relatively small (≈0.05–0.15 kcal/mol). Another key difference may arise from T487 → N501 substitution, where the binding energy loss of 2.48 kcal/mol for N501A is markedly larger than the change of 1.32 kcal/mol for T487A mutation in SARS-CoV (Figure 10A,B). More importantly, the binding energy hotspots of SARS-CoV-2 can be better supported by structurally proximal residues that collectively contribute to the broader binding interface as compared to SARS-CoV-RBD. The key hotspot Q493 in the central segment of the interface is supported by residues K417, Y453, L455, and F456 that contribute significantly to the binding energy of SARS-CoV-2 (Figure 10B–D). Another notable difference is between F456 in SARS-CoV-2 and corresponding L443 in SARS-CoV. The binding energy difference of 2.18 kcal/mol for F456A may be contrasted to only 1.06 kcal/mol for L443A mutation in SARS-CoV. The more favorable interactions could be also seen for the residue stretch 501-NGVGY-505 surrounding N501 hotspot in SARS-CoV-2 as compared to corresponding region 487-TGIGY-491 in SARS-CoV (Figure 10A,B). The important conclusion of our analysis is that the binding energy contributions appear to be distributed more broadly and densely across the SARS-CoV-2 RBD binding interface. In some contrast, the binding energy of the SARS-CoV RBD may be determined by several sparsely localized patches of the interface residues.

**Figure 11:**
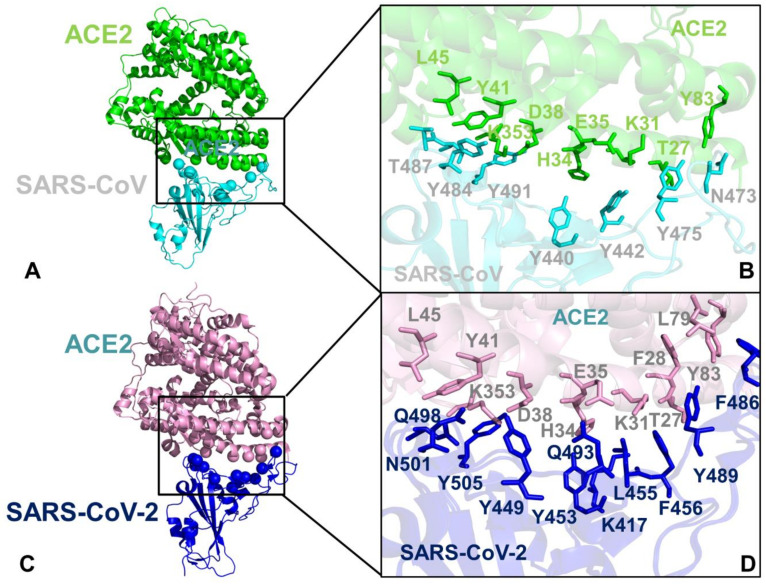
Structural mapping of the binding energy hotspots in the SARS-CoV-RBD and SARS-Cov-2-RBD complexes with ACE2. (**A**) A general overview of the SARS-CoV-RBD complex with ACE2. SARS-CoV-RBD is shown in cyan ribbons and ACE2 is in green ribbons. The binding energy hotspots that experience significant loss of binding energy upon alanine mutations are shown in cyan spheres for SARS-CoV-RBD and in green spheres for ACE2. (**B**) A close-up of these binding energy hotspots in the SARS-CoV-RBD complex with ACE2. The SARS-CoV-RBD residues are annotated and shown in cyan sticks, ACE2 hotspot residues are in green sticks. (**C**) A general overview of the SARS-CoV-2-RBD complex with ACE2. SARS-Cov-2-RBD is shown in blue ribbons and ACE2 is in pink ribbons. The binding energy hotspots that experience significant loss of binding energy upon alanine mutations are shown in blue spheres for SARS-CoV-2-RBD and in pink spheres for ACE2. (**D**) A close-up of the binding energy hotspots in the SARS-CoV-2-RBD complex with ACE2. The SARS-CoV-RBD residues are annotated and shown in blue sticks, ACE2 hotspot residues are in pink sticks.

Our results also provided some useful insights to the binding mechanism based on rearrangements of two virus-binding hotspots defined by a salt bridge between K31 and E35 and a salt bridge between K353 and D38 in the SARS-CoV-RBD (Figure 11A,B). According to the performed energetic analysis, the network of SARS-CoV-2 RBM interactions formed by the middle segment of the interface (K417, Y453, L455, F456, and Q493) with K31 and E35 is vastly different and considerably stronger than the corresponding interactions made in the SARS-CoV complex (Figure 10). The loss of salt bridge K31-E35 broken in the SARS-CoV-2 RBM complex is counterbalanced not only by the hydrogen bonding interactions with Q493 but is also reinforced through an extensive contact network with other RBM residues (Figure 11C,D). The energetic analysis revealed that the binding energy hotspots on ACE2 corresponded to residues T27, F28, K31, H34, Y41, and Y83 displaying consistently larger mutation-induced free energy changes in the SARS-CoV-2 RBM complex (Figure 10B–D). Hence, the virus-binding hotspot on ACE2 formed by K31, H34, and E35 may be an important driving force of binding selectivity for SARS-CoV-2 RBD (Figure 11C,D). Another hotspot on ACE2 is K353-D38 salt bridge that is surrounded by hydrophobic walls formed by Y41 and D37 residues [67]. This hotspot is supported by T487 and Y491 in SARS-CoV-RBD (Figure 11A,B) and by N501 and Y505 in SARS-CoV-2-RBD (Figure 11C,D). Although this hotspot is important for recognition of spike protein, the binding energy contributions of the corresponding residues in the SARS-CoV-RBD (Figure 10A) and SARS-CoV-2 RBD (Figure 10B–D) are relatively moderate and similar, suggesting that this hotspot may be less critical for driving binding selectivity of SARS-CoV-2-RBD.

We also conducted a systematic alanine scanning of the RBD spike protein residues in which FoldX energy function was used to estimate protein stability changes in all studied complexes (Figure S3). As expected, the protein stability scanning profiles were generally similar for SARS-CoV (Figure S3A) and SARS-CoV-2 residues (Figure S3B–D). However, the results also indicated the greater stability of the binding interface residues from RBM, displaying larger individual peaks and also showing stronger stability of the interfacial residues surrounding the key hotspots (Figure S3B). Collectively, our results pointed to several interesting trends: (a) the strongest binding energy hotspots are conserved between SARS-CoV and SARS-CoV-2 proteins (Y440 → Y453, Y475 → Y489 and Y491 → Y505); (b) the largest energetic differences may be attributed to the key energy hotpot residues Q493 and N501 in SARS-CoV-2 RBD; (c) SARS-CoV-2 binding selectivity preferences could be strongly influenced by the virus-binding hotspot K31 and H34 in the middle of the interface through an extensive interaction network with K417, Y453, L455, F456, and Q493; (d) the binding energy contributions are broadly and densely distributed across many interfacial residues of the SARS-CoV-2 RBD supporting the key hotspot positions. The results suggested that the differences in the binding affinities of SARS spike proteins may arise from a cumulative contribution of small energetic changes distributed across the entire interface rather than from localized changes in several binding energy hotspots. 

## 3. Materials and Methods

### 3.1. Sequence Conservation and Coevolutionary Analyses

Sequence conservation for SARS-CoV spike proteins and ACE2 enzymes were estimated using ConSurf approach [38,39] by computing the residue-based conservation score profiles that measure evolutionary conservation, and the low score values are associated with the most conserved position in the protein. Multiple sequence alignment (MSA) of 8264 SARS-CoV-2 sequences retrieved from NCBI virus was obtained using MAFFT approach [68]. In this alignment, 4311 homologues were obtained from UNIPROT database and UNIREF90 [69]. We also employed the Kullback–Leibler (KL) sequence conservation score KLConsScore using the MSA profile generated for the SARS-CoV Spike glycoprotein and ACE2 proteins. MSA profiles were obtained from Pfam database of protein families (P59594, SPIKE_CVHSA, and CoV_S1_C, PF19209) and for ACE2 enzyme (Peptidase_M2, PF01401) [70,71,72] generated by hidden Markov models. For ACE2 proteins 2560 sequences from 758 species were used to generate MSA profiles. Of these, 2787 homologues passed the thresholds. The calculations were conducted on 300 hits (query included), sampled from the unique hits. The KL conservation is calculated as

(1) $$KLConsScore_{i}=\sum_{i=1}^{N}\ln\frac{P(i)}{Q(i)}$$

Here, P(i) is the frequency of amino acid *i* in that position and Q(i) is the background frequency of the amino acid in nature calculated using an amino acids background frequency distribution obtained from the UniProt database [73]. Mutual Information (*MI*) analysis and computations were done using by MISTIC approach yielding the coevolutionary relationships between pairs of positions in the SARS spike proteins and ACE2 proteins [43,44]. To evaluate coevolutionary couplings, the covariance metric based on *MI* calculations was adjusted by the average product correction (APC) [45,46,47]. Using coevolutionary residue matrices computations [59] we reconstructed a network of coevolutionary residues with nodes representing residues and the inter-node links corresponded to MI-derived coevolutionary couplings between these residues. Cumulative mutual information (*cMI*) parameter evaluates the shared mutual information between a given residue and other protein residues, and proximity-based mutual information parameter (*pMI*) measures the mutual information shared by a given residue with the structurally close neighboring nodes. *cMI* was calculated as the sum of *MI* values above a defined threshold (*t* = 6.0) for every pair that contains a given residue:

(2) $$cMI_{x}=\sum_{y,MI(x,y)>t}MI_{\left(x,y\right)}$$

*pMI* score was computed using the following formula where the local residue environment was averaged over MD simulations and a threshold distance *t* = 5 Å defined residue proximity:

(3) $$pMI_{x}=\frac{1}{N}\sum_{d(x,y),t}cMI_{\left(x,y\right)}$$

### 3.2. Coarse-Grained Molecular Simulations

We employed coarse-grained (CG) CABS model in which CG representation of amino acid residues is reduced to four united atoms [74,75,76,77,78]. The amino acid residues are represented by main-chain α-carbons, β-carbons, the center of mass of side chains, and another pseudo-atom placed in the center of the Cα-Cα pseudo-bond. The sampling scheme of the CABS model allows for replica-exchange simulations and is based on Monte Carlo dynamics and involves a sequence of local moves of individual amino acids in the protein structure as well as moves of small fragments [74,75,76]. In each simulation, the total number of cycles was set to 10,000 and the number of cycles between trajectory frames was 100. A total of 100 multiple independent CG simulations were performed for the all unbound and bound SARS-CoV-RBD and SARS-CoV-2-RBD structures. Multiple independent simulations produced a total of 1,000,000 samples for each studied system and the total number of saved models in the trajectory used for analysis was 10,000. CABS dynamics protocol has been previously validated on a variety of protein systems, yielding accurate dynamics trajectories for long-time processes [74,75,76,77,78]. Although the time unit of such dynamics cannot be rigorously defined, the implemented protocol is expected to mimic the microsecond time scale of conformational changes and can be used for direct comparative analysis with atomistic MD simulations. CABS-flex standalone program was employed for the analysis of the CG simulations and all-atom reconstruction [78]. The simulated unbound forms included the crystal structure of SARS spike protein RBD (pdb id 2GHV) [79] and the crystal structure of the human ACE2 enzyme [48] (pdb id 1R42). The following complexes were simulated and analyzed: the crystal structures of SARS-CoV-RBD complexed with ACE2 receptor (pdb id 2AJF) [14,24], and the crystal structures of SARS-CoV-2-RBD bound with ACE2 (pdb id 6M0J, 6VW1, 6M17) [24,25,26]. All structures were obtained from the Protein Data Bank [80,81]. The CG conformational ensembles of the SARS-CoV-RBD and SARS-CoV-2-RBD structures were then subjected to all-atom reconstruction using PULCHRA method [82] and CG2AA tool [83]. The all-atom conformations were additionally optimized using the 3Drefine method [84] that utilizes atomic-level energy minimization with a composite physics and knowledge-based force fields.

### 3.3. Structure Preparation and All-Atom Molecular Dynamics Simulations

All-atom 1µs MD simulations were performed for all studied protein structures. All crystallographic water molecules and other heteroatoms including zinc were initially removed. The zinc-binding site is located near the bottom of the cleft of subdomain I of ACE2 and more than 20 Å away from the intermolecular binding interface with SARS-CoV/SARS-CoV-2 RBD proteins. Furthermore, previous studies have established that zinc does not stabilize ACE2 protein structure since the native and apo-enzymes are equally susceptible to heat denaturation [85]. In addition, it was shown that metal replacements mainly affect catalytic activity while a negligible change on ACE2 binding and dissociation constant can be observed [85,86]. The hydrogen atoms and missing residues were initially assigned using the WHATIF program [87]. Hydrogen atoms were initially incorporated and the protonation states on protein residues were generated with the WHATIF program and then refined by the H++ web server [88]. The structures were further preprocessed through the Protein Preparation Wizard (Schrödinger, LLC, New York, NY) and included the check of bond order, assignment and adjustment of ionization states, formation of disulphide bonds, removal of crystallographic water molecules and cofactors, capping of the termini, assignment of partial charges, and addition of possible missing atoms and side chains that were not assigned in the initial processing with the WHATIF program. These preparation steps were consistent with the recent MD simulations of these systems [66]. Additionally, and for comparison, the missing loops in the structures were also reconstructed using template-based loop prediction approaches ModLoop [89] and ArchPRED server [90]. The unresolved structural segments were modeled and refined using the program MODELLER [91]. The crystal structures of the unbound SARS-CoV and SARS-CoV-2 Spike RBD proteins were initially placed in orthorhombic boxes of size 120 × 120 × 120 Å and solvated with simple point charge model. The crystal structures of the SARS-CoV/SARS-CoV-2 RBD complexes were simulated in a box size of 85 × 85 × 85 Å with buffering distance of 12 Å. Assuming normal charge states of ionizable groups corresponding to pH = 7, sodium (Na+) and chloride (Cl-) counter-ions were added to achieve charge neutrality and a salt concentration of 0.15 M NaCl was maintained. All Na^+^ and Cl^−^ ions were placed at least 8 Å away from any protein atoms and from each other. MD simulations were performed using CHARMM22 force field [92] with the explicit TIP3P water model [93] as implemented in the NAMD software package [94]. The following protocol preceded the production stage of MD simulations. Long-range non-bonded van der Waals interactions were computed using an atom-based cutoff of 12 Å with switching van der Waals potential beginning at 10 Å. Long-range electrostatic interactions were calculated using the particle mesh Ewald method [95] with a real space cut-off of 1.0 nm and a fourth order (cubic) interpolation. SHAKE method was used to constrain all bonds associated with hydrogen atoms. An integration step size of 2 fs was used, and production simulation trajectories were saved every 100 ps. Simulations were run using a leap-frog integrator with a 2 fs integration time step and with Verlet buffered lists (target energy drift of 0.005 kJ mol^−1^ ns^−1^ per atom). The neighbor list update frequency was set to every 50 steps. Energy minimization after addition of solvent and ions was carried out using the steepest descent method for 100,000 steps. All atoms of the complex were first restrained at their crystal structure positions with a force constant of 10 Kcal mol^−1^ Å^−2^. Equilibration was done in steps by gradually increasing the system temperature in steps of 20K starting from 10K until 310 K and at each step 1ns equilibration was run keeping a restraint of 10 Kcal mol-1 Å-2 on the protein alpha carbons (C_α_). After the restrains on the protein atoms were removed, a series of short equilibration simulations for 500 ps in NVT and 500 ps in the NPT ensembles were performed. Finally, the system was equilibrated for additional 10 ns. An NPT production simulation was run on the equilibrated structures for 1µs keeping the temperature at 310 K and constant pressure (1 atm). In simulations, the Nose–Hoover thermostat [96] and isotropic Martyna–Tobias–Klein barostat [97] were used to maintain the temperature at 310 K and pressure at 1 atm, respectively.

### 3.4. Protein Stability Analysis and Binding Free Energy Calculations

Several different approaches were employed to compute (a) protein binding free energy changes induced by alanine mutations of the binding site residues; (b) mutational sensitivity and protein stability changes using systematic alanine scanning of protein residues. The FoldX approach with the all-atom representation of protein structure [98] was used to conduct alanine scanning of the residues and evaluate protein stability changes in the complexes. We utilized a graphical user interface for the FoldX calculations [99,100]. The protein stability ΔΔG changes were computed by averaging the results of computations over 1000 samples obtained from MD simulation trajectories [101,102]. The binding free energy of protein–protein complex can be expressed as the difference in the folding free energy of the complex and folding free energies of the two protein binding partners:

(4) $$\Delta G_{bind}=G^{com}-G^{A}-G^{B}$$

The change of the binding energy due to a mutation was calculated then as the following:

(5) $$\Delta \Delta G_{bind}=\Delta G_{bind}^{mut}-\Delta G_{bind}^{wt}{{}_{}}^{}$$

The binding free energies were also calculated using BeAtMuSiC approach that is based on statistical potentials and describe the pairwise inter-residue distances, backbone torsion angles and solvent accessibilities, and considers the effect of the mutation on the strength of the interactions at the interface and on the overall stability of the complex [103,104,105]. We leveraged rapid calculations based on statistical potentials to compute the ensemble-averaged binding free energy changes using equilibrium samples from MD trajectories. The binding free energy changes were computed by averaging the results over 1000 equilibrium samples for each system.

## 4. Conclusions

In this work, we combined sequence and coevolutionary analyses with coarse-grained and all-atom MD simulations of the SARS-CoV-RBD spike proteins in the unbound and bound proteins to dissect molecular determinants of binding mechanisms and drivers of preferential binding for SARS-CoV-2 RBD protein. The analysis of structural and thermodynamic factors underlying the binding mechanism was done by leveraging atomistic simulations of protein dynamics and modeling of coevolutionary networks that suggested several important drivers of binding and signal transmission. We also established that the binding interface between SARS-CoV-RBD and ACE2 proteins can involve contributions of both conserved and more variable positions that are broadly distributed and may cooperate in the binding process. By exploring the molecular basis of the virus entry mechanism through the lens of structure, dynamics, and coevolution, we found that structurally stable and conserved sites in the central segment of the binding interface can regulate coevolutionary couplings and determine the SARS-CoV-RBD recognition mode. These sites anchor networks of highly coevolving residues in SARS-CoV-RBD and ACE2 that tend to be located in the mobile loops of the binding interface. We argue that these flexible and moderately conserved residues could act as flexible carriers of allosteric signals between interacting proteins. The important result of the conformational dynamics is the evidence of a much broader and consistent stabilization across the entire RBM interface with ACE2 for the SARS-CoV-2-RBD protein. Ensemble-based mutational scanning and energetic analysis has revealed a cooperative effect of multiple residues in SARS-CoV-2-RBD in the binding process as binding energy contributions are broadly distributed across many interfacial residues supporting the key hotspot positions. The central findings of this study revealing that the binding energetics of SARS-CoV-2-RBD can be broadly distributed across the entire interface with ACE2 in supporting the hotspot positions highlighted drug discovery challenges that would likely require inhibition of large and adaptive protein–protein interfaces in addition to targeting the discovered hotspots responsible for virus entry. While the current therapeutic strategies against SARS-CoV-2 proteins involve a variety of protein targets and robust drug repurposing pipelines, the effective agents targeting SARS-CoV-2 binding interfaces may require SAR-by-NMR approaches and elaborate drug design protocols to block a highly adaptive interface involving cooperative contributions of multiple sites. The findings from this study suggest that the binding mechanism of SARS-CoV-2 RBD may operate through dynamic redistribution of the interface residues beyond several hotspots, suggesting that exploring potential allosteric sites and modulators that can alter dynamics in the SARS-CoV-2 complexes with ACE2 could be a useful strategy for drug design. The next breakthroughs in the discovery of effective drugs of SARS-CoV-2 may ultimately require synergistic approaches that involve combinations of targeted and allosteric molecules affecting the recognition events, the molecular basis of binding mechanisms, and virus transmission.

## Figures and Tables

**Table 1:** The evolutionary conserved hotspot residues determined with ConSurf and KL scores.

ConSurf Hotspots	Consurf Score	KL Hotspots	KL Score
K390	−0.390	K390	1.12
V404	−0.367	V404	1.07
G434	−0.505	Y436	1.45
Y440	−0.705	Y440	1.19
N473	−0.467	N473	1.51
Y475	−0.705	Y475	1.63
Y481	473	Y481	0.72

**Table 2:** The pMI and cMI hotspot residues for SARS-CoV-RBD and SARS-CoV-2 RBD proteins.

SARS-CoV pMI Hotspot Sites	SARS-CoV-2 pMI Hotspot Sites	SARS-CoV cMI Hotspot Sites	SARS-CoV-2 cMI Hotspot Sites
Y436	Y449	L472	F486
Y440	Y451	N479	Q493
Y475	Y489	T487	N501
W476	F490	T433	G446
Y481	Y495	T468	N481
Y484	Q498	P470	E484
N473	N487	Y484	Q498

## Supplementary Materials

Supplementary materials can be found at https://www.mdpi.com/1422-0067/21/21/8268/s1.

## References

[1] Li Q., Guan X., Wu P., Wang X., Zhou L., Tong Y., Ren R., Leung K.S.M., Lau E.H.Y., Wong J.Y. (2020). Early Transmission Dynamics in Wuhan, China, of Novel Coronavirus-Infected Pneumonia. N. Engl. J. Med..

[2] Wang C., Horby P.W., Hayden F.G., Gao G.F. (2020). A novel coronavirus outbreak of global health concern. Lancet.

[3] Yi Y., Lagniton P.N.P., Ye S., Li E., Xu R.H. (2020). COVID-19: What has been learned and to be learned about the novel coronavirus disease. Int. J. Biol. Sci..

[4] Wu A., Peng Y., Huang B., Ding X., Wang X., Niu P., Meng J., Zhu Z., Zhang Z., Wang J. (2020). Genome Composition and Divergence of the Novel Coronavirus (2019-nCoV) Originating in China. Cell Host Microbe.

[5] de Wit E., van Doremalen N., Falzarano D., Munster V.J. (2016). SARS and MERS: Recent insights into emerging coronaviruses. Nat. Rev. Microbiol..

[6] Huang C., Wang Y., Li X., Ren L., Zhao J., Hu Y., Zhang L., Fan G., Xu J., Gu X. (2020). Clinical features of patients infected with 2019 novel coronavirus in Wuhan, China. Lancet.

[7] Indwiani Astuti Y. (2020). Severe Acute Respiratory Syndrome Coronavirus 2 (SARS-CoV-2): An overview of viral structure and host response. Diabetes Metab. Syndr..

[8] Schoeman D., Fielding B.C. (2019). Coronavirus envelope protein: Current knowledge. Virol. J..

[9] Tai W., He L., Zhang X., Pu J., Voronin D., Jiang S., Zhou Y., Du L. (2020). Characterization of the receptor-binding domain (RBD) of 2019 novel coronavirus: Implication for development of RBD protein as a viral attachment inhibitor and vaccine. Cell. Mol. Immunol..

[10] Hoffmann M., Kleine-Weber H., Schroeder S., Krüger N., Herrler T., Erichsen S., Schiergens T.S., Herrler G., Wu N.H., Nitsche A. (2020). SARS-CoV-2 Cell Entry Depends on ACE2 and TMPRSS2 and Is Blocked by a Clinically Proven Protease Inhibitor. Cell.

[11] Zhou P., Yang X.L., Wang X.G., Hu B., Zhang L., Zhang W., Si H.R., Zhu Y., Li B., Huang C.L. (2020). A pneumonia outbreak associated with a new coronavirus of probable bat origin. Nature.

[12] Lu R., Zhao X., Li J., Niu P., Yang B., Wu H., Wang W., Song H., Huang B., Zhu N. (2020). Genomic characterisation and epidemiology of 2019 novel coronavirus: Implications for virus origins and receptor binding. Lancet.

[13] Tortorici M.A., Veesler D. (2019). Structural insights into coronavirus entry. Adv. Virus Res..

[14] Wang Q., Zhang Y., Wu L., Niu S., Song C., Zhang Z., Lu G., Qiao C., Hu Y., Yuen K.Y. (2020). Structural and Functional Basis of SARS-CoV-2 Entry by Using Human ACE2. Cell.

[15] Wan Y., Shang J., Graham R., Baric R.S., Li F. (2020). Receptor Recognition by the Novel Coronavirus from Wuhan: An Analysis Based on Decade-Long Structural Studies of SARS Coronavirus. J. Virol..

[16] Walls A.C., Park Y.J., Tortorici M.A., Wall A., McGuire A.T., Veesler D. (2020). Structure, Function, and Antigenicity of the SARS-CoV-2 Spike Glycoprotein. Cell.

[17] Gui M., Song W., Zhou H., Xu J., Chen S., Xiang Y., Wang X. (2017). Cryo-electron microscopy structures of the SARS-CoV spike glycoprotein reveal a prerequisite conformational state for receptor binding. Cell Res..

[18] Walls A.C., Xiong X., Park Y.J., Tortorici M.A., Snijder J., Quispe J., Cameroni E., Gopal R., Dai M., Lanzavecchia A. (2019). Unexpected Receptor Functional Mimicry Elucidates Activation of Coronavirus Fusion. Cell.

[19] Yuan Y., Cao D., Zhang Y., Ma J., Qi J., Wang Q., Lu G., Wu Y., Yan J., Shi Y. (2017). Cryo-EM structures of MERS-CoV and SARS-CoV spike glycoproteins reveal the dynamic receptor binding domains. Nat. Commun..

[20] Wrapp D., Wang N., Corbett K.S., Goldsmith J.A., Hsieh C.L., Abiona O., Graham B.S., McLellan J.S. (2020). Cryo-EM structure of the 2019-nCoV spike in the prefusion conformation. Science.

[21] Li F., Li W., Farzan M., Harrison S.C. (2005). Structure of SARS coronavirus spike receptor-binding domain complexed with receptor. Science.

[22] Chakraborti S., Prabakaran P., Xiao X., Dimitrov D.S. (2005). The SARS coronavirus S glycoprotein receptor binding domain: Fine mapping and functional characterization. Virol. J..

[23] He Y., Lu H., Siddiqui P., Zhou Y., Jiang S. (2005). Receptor-binding domain of severe acute respiratory syndrome coronavirus spike protein contains multiple conformation-dependent epitopes that induce highly potent neutralizing antibodies. J. Immunol..

[24] Lan J., Ge J., Yu J., Shan S., Zhou H., Fan S., Zhang Q., Shi X., Wang Q., Zhang L. (2020). Structure of the SARS-CoV-2 spike receptor-binding domain bound to the ACE2 receptor. Nature.

[25] Shang J., Ye G., Shi K., Wan Y., Luo C., Aihara H., Geng Q., Auerbach A., Li F. (2020). Structural basis of receptor recognition by SARS-CoV-2. Nature.

[26] Yan R., Zhang Y., Li Y., Xia L., Guo Y., Zhou Q. (2020). Structural basis for the recognition of SARS-CoV-2 by full-length human ACE2. Science.

[27] Starr T.N., Greaney A.J., Hilton S.K., Crawford K.H.D., Navarro M.J., Bowen J.E., Tortorici M.A., Walls A.C., Veesler D., Bloom J.D. (2020). Deep mutational scanning of SARS-CoV-2 receptor binding domain reveals constraints on folding and ACE2 binding. Cell.

[28] Procko E. (2020). The sequence of human ACE2 is suboptimal for binding the S spike protein of SARS coronavirus 2. bioRxiv.

[29] Brielle E.S., Schneidman-Duhovny D., Linial M. (2020). The SARS-CoV-2 Exerts a Distinctive Strategy for Interacting with the ACE2 Human Receptor. Viruses.

[30] Spinello A., Saltalamacchia A., Magistrato A. (2020). Is the Rigidity of SARS-CoV-2 Spike Receptor-Binding Motif the Hallmark for Its Enhanced Infectivity? Insights from All-Atom Simulations. J. Phys. Chem. Lett..

[31] Chen Y., Guo Y., Pan Y., Zhao Z.J. (2020). Structure analysis of the receptor binding of 2019-nCoV. Biochem. Biophys. Res. Commun..

[32] Veeramachaneni G.K., Thunuguntla V.B.S.C., Bobbillapati J., Bondili J.S. (2020). Structural and simulation analysis of hotspot residues interactions of SARS-CoV 2 with human ACE2 receptor. J. Biomol. Struct. Dyn..

[33] Wang Y., Liu M., Gao J. (2020). Enhanced receptor binding of SARS-CoV-2 through networks of hydrogen-bonding and hydrophobic interactions. Proc. Natl. Acad. Sci. USA.

[34] Casalino L., Gaieb Z., Goldsmith J.A., Hjorth C.K., Dommer A.C., Harbison A.M., Fogarty C.A., Barros E.P., Taylor B.C., McLellan J.S. (2020). Beyond Shielding: The Roles of Glycans in the SARS-CoV-2 Spike Protein. ACS Cent. Sci..

[35] Kalathiya U., Padariya M., Mayordomo M., Lisowska M., Nicholson J., Singh A., Baginski M., Fahraeus R., Carragher N., Ball K. (2020). Highly Conserved Homotrimer Cavity Formed by the SARS-CoV-2 Spike Glycoprotein: A Novel Binding Site. J. Clin. Med..

[36] Di Paola L., Hadi-Alijanvand H., Song X., Hu G., Giuliani A. (2020). The Discovery of a Putative Allosteric Site in the SARS-CoV-2 Spike Protein Using an Integrated Structural/Dynamic Approach. J. Proteome Res..

[37] Verkhivker G.M. (2020). Molecular Simulations and Network Modeling Reveal an Allosteric Signaling in the SARS-CoV-2 Spike Proteins. J. Proteome Res..

[38] Ashkenazy H., Abadi S., Martz E., Chay O., Mayrose I., Pupko T., Ben-Tal N. (2016). ConSurf 2016: An improved methodology to estimate and visualize evolutionary conservation in macromolecules. Nucleic Acids Res..

[39] Ben Chorin A., Masrati G., Kessel A., Narunsky A., Sprinzak J., Lahav S., Ashkenazy H., Ben-Tal N. (2020). ConSurf-DB: An accessible repository for the evolutionary conservation patterns of the majority of PDB proteins. Protein Sci..

[40] Jaimes J.A., André N.M., Chappie J.S., Millet J.K., Whittaker G.R. (2020). Phylogenetic Analysis and Structural Modeling of SARS-CoV-2 Spike Protein Reveals an Evolutionary Distinct and Proteolytically Sensitive Activation Loop. J. Mol. Biol..

[41] Shah M., Ahmad B., Choi S., Woo H.G. (2020). Sequence variation of SARS-CoV-2 spike protein may facilitate stronger interaction with ACE2 promoting high infectivity. Res. Sq..

[42] Armijos-Jaramillo V., Yeager J., Muslin C., Perez-Castillo Y. (2020). SARS-CoV-2, an evolutionary perspective of interaction with human ACE2 reveals undiscovered amino acids necessary for complex stability. Evol. Appl..

[43] Marino Buslje C., Teppa E., Di Domenico T., Delfino J.M., Nielsen M. (2010). Networks of high mutual information define the structural proximity of catalytic sites: Implications for catalytic residue identification. PLoS Comput. Biol..

[44] Simonetti F.L., Teppa E., Chernomoretz A., Nielsen M., Marino Buslje C. (2013). MISTIC: Mutual information server to infer coevolution. Nucleic Acids Res..

[45] Tillier E.R.M., Lui T.W.H. (2003). Using multiple interdependency to separate functional from phylogenetic correlations in protein alignments. Bioinformatics.

[46] Martin L.C., Gloor G.B., Dunn S.D., Wahl L.M. (2005). Using information theory to search for co-evolving residues in proteins. Bioinformatics.

[47] Dunn S.D., Wahl L.M., Gloor G.B. (2008). Mutual information without the influence of phylogeny or entropy dramatically improves residue contact prediction. Bioinformatics.

[48] Towler P., Staker B., Prasad S.G., Menon S., Tang J., Parsons T., Ryan D., Fisher M., Williams D., Dales N.A. (2004). ACE2 X-ray structures reveal a large hinge-bending motion important for inhibitor binding and catalysis. J. Biol. Chem..

[49] Macours N., Poels J., Hens K., Francis C., Huybrechts R. (2004). Structure, evolutionary conservation, and functions of angiotensin- and endothelin-converting enzymes. Int. Rev. Cytol..

[50] Warner F.J., Smith A.I., Hooper N.M., Turner A.J. (2004). Angiotensin-converting enzyme-2: A molecular and cellular perspective. Cell. Mol. Life Sci..

[51] Kuhn J.H., Li W., Choe H., Farzan M. (2004). Angiotensin-converting enzyme 2: A functional receptor for SARS coronavirus. Cell. Mol. Life Sci..

[52] Vangone A., Bonvin A.M. (2015). Contacts-based prediction of binding affinity in protein-protein complexes. Elife.

[53] Xue L.C., Rodrigues J.P., Kastritis P.L., Bonvin A.M., Vangone A. (2016). PRODIGY: A web server for predicting the binding affinity of protein-protein complexes. Bioinformatics.

[54] Lee B.C., Park K., Kim D. (2008). Analysis of the residue-residue coevolution network and the functionally important residues in proteins. Proteins.

[55] Chakrabarti S., Panchenko A.R. (2009). Coevolution in defining the functional specificity. Proteins.

[56] Chakrabarti S., Panchenko A.R. (2010). Structural and functional roles of coevolved sites in proteins. PLoS ONE.

[57] Teppa E., Wilkins A.D., Nielsen M., Buslje C.M. (2012). Disentangling evolutionary signals: Conservation, specificity determining positions and coevolution. Implication for catalytic residue prediction. BMC Bioinform..

[58] Jeon J., Nam H.J., Choi Y.S., Yang J.S., Hwang J., Kim S. (2011). Molecular evolution of protein conformational changes revealed by a network of evolutionarily coupled residues. Mol. Biol. Evol..

[59] Stetz G., Verkhivker G.M. (2017). Computational Analysis of Residue Interaction Networks and Coevolutionary Relationships in the Hsp70 Chaperones: A Community-Hopping Model of Allosteric Regulation and Communication. PLoS Comput. Biol..

[60] Tse A., Verkhivker G.M. (2015). Molecular Determinants Underlying Binding Specificities of the ABL Kinase Inhibitors: Combining Alanine Scanning of Binding Hot Spots with Network Analysis of Residue Interactions and Coevolution. PLoS ONE.

[61] Stetz G., Tse A., Verkhivker G.M. (2018). Dissecting Structure-Encoded Determinants of Allosteric Cross-Talk between Post-Translational Modification Sites in the Hsp90 Chaperones. Sci. Rep..

[62] Popovych N., Sun S., Ebright R.H., Kalodimos C.G. (2006). Dynamically driven protein allostery. Nat. Struct. Mol. Biol..

[63] Tzeng S.R., Kalodimos C.G. (2009). Dynamic activation of an allosteric regulatory protein. Nature.

[64] Huang C., Kalodimos C.G. (2017). Structures of Large Protein Complexes Determined by Nuclear Magnetic Resonance Spectroscopy. Annu. Rev. Biophys..

[65] Jiang Y., Kalodimos C.G. (2017). NMR Studies of Large Proteins. J. Mol. Biol..

[66] Ali A., Vijayan R. (2020). Dynamics of the ACE2-SARS-CoV-2/SARS-CoV spike protein interface reveal unique mechanisms. Sci. Rep..

[67] Wu K., Chen L., Peng G., Zhou W., Pennell C.A., Mansky L.M., Geraghty R.J., Li F. (2011). A virus-binding hot spot on human angiotensin-converting enzyme 2 is critical for binding of two different coronaviruses. J. Virol..

[68] Rozewicki J., Li S., Amada K.M., Standley D.M., Katoh K. (2019). MAFFT-DASH: Integrated protein sequence and structural alignment. Nucleic Acids Res..

[69] Suzek B.E., Wang Y., Huang H., McGarvey P.B., Wu C.H. (2015). UniRef clusters: A comprehensive and scalable alternative for improving sequence similarity searches. Bioinformatics.

[70] Finn R.D., Bateman A., Clements J., Coggill P., Eberhardt R.Y., Eddy S.R., Heger A., Hetherington K., Holm L., Mistry J. (2014). Pfam: The protein families database. Nucleic Acids Res..

[71] El-Gebali S., Mistry J., Bateman A., Eddy S.R., Luciani A., Potter S.C., Qureshi M., Richardson L.J., Salazar G.A., Smart A. (2019). The Pfam protein families database in 2019. Nucleic Acids Res..

[72] Finn R.D., Miller B.L., Clements J., Bateman A. (2014). iPfam: A database of protein family and domain interactions found in the Protein Data Bank. Nucleic Acids Res..

[73] Wu C.H., Apweiler R., Bairoch A., Natale D.A., Barker W.C., Boeckmann B., Ferro S., Gasteiger E., Huang H., Lopez R. (2006). The Universal Protein Resource (UniProt): An expanding universe of protein information. Nucleic Acids Res..

[74] Kolinski A. (2004). Protein modeling and structure prediction with a reduced representation. Acta Biochim. Pol..

[75] Kmiecik S., Gront D., Kolinski M., Wieteska L., Dawid A.E., Kolinski A. (2016). Coarse-Grained Protein Models and Their Applications. Chem. Rev..

[76] Kmiecik S., Kouza M., Badaczewska-Dawid A.E., Kloczkowski A., Kolinski A. (2018). Modeling of Protein Structural Flexibility and Large-Scale Dynamics: Coarse-Grained Simulations and Elastic Network Models. Int. J. Mol. Sci..

[77] Ciemny M.P., Badaczewska-Dawid A.E., Pikuzinska M., Kolinski A., Kmiecik S. (2019). Modeling of disordered protein structures using monte carlo simulations and knowledge-based statistical force fields. Int. J. Mol. Sci..

[78] Kurcinski M., Oleniecki T., Ciemny M.P., Kuriata A., Kolinski A., Kmiecik S. (2019). CABS-flex standalone: A simulation environment for fast modeling of protein flexibility. Bioinformatics.

[79] Hwang W.C., Lin Y., Santelli E., Sui J., Jaroszewski L., Stec B., Farzan M., Marasco W.A., Liddington R.C. (2006). Structural basis of neutralization by a human anti-severe acute respiratory syndrome spike protein antibody, 80R. J. Biol. Chem..

[80] Berman H.M., Westbrook J., Feng Z., Gilliland G., Bhat T.N., Weissig H., Shindyalov I.N., Bourne P.E. (2000). The Protein Data Bank. Nucleic Acids Res..

[81] Rose P.W., Prlic A., Altunkaya A., Bi C., Bradley A.R., Christie C.H., Costanzo L.D., Duarte J.M., Dutta S., Feng Z. (2017). The RCSB protein data bank: Integrative view of protein, gene and 3D structural information. Nucleic Acids Res..

[82] Rotkiewicz P., Skolnick J. (2008). Fast procedure for reconstruction of full-atom protein models from reduced representations. J. Comput. Chem..

[83] Lombardi L.E., Marti M.A., Capece L. (2016). CG2AA: Backmapping protein coarse-grained structures. Bioinformatics.

[84] Bhattacharya D., Nowotny J., Cao R., Cheng J. (2016). 3Drefine: An interactive web server for efficient protein structure refinement. Nucleic Acids Res..

[85] Bünning P., Riordan J.F. (1985). The functional role of zinc in angiotensin converting enzyme: Implications for the enzyme mechanism. J. Inorg. Biochem..

[86] Bernstein K.E., Ong F.S., Blackwell W.L., Shah K.H., Giani J.F., Gonzalez-Villalobos R.A., Shen X.Z., Fuchs S., Touyz R.M. (2012). A modern understanding of the traditional and nontraditional biological functions of angiotensin-converting enzyme. Pharmacol. Rev..

[87] Hekkelman M.L., Te Beek T.A.H., Pettifer S.R., Thorne D., Attwood T.K., Vriend G. (2010). WIWS: A Protein Structure Bioinformatics Web Service Collection. Nucleic Acids Res..

[88] Anandakrishnan R., Aguilar B., Onufriev A.V. (2012). H++ 3.0: Automating pK prediction and the preparation of biomolecular structures for atomistic molecular modeling and simulations. Nucleic Acids Res..

[89] Fiser A., Sali A. (2003). ModLoop: Automated Modeling of Loops in Protein Structures. Bioinformatics.

[90] Fernandez-Fuentes N., Zhai J., Fiser A. (2006). ArchPRED: A Template Based Loop Structure Prediction Server. Nucleic Acids Res..

[91] Marti-Renom M.A., Stuart A.C., Fiser A., Sanchez R., Melo F., Sali A. (2000). Comparative protein structure modeling of genes and genomes. Annu. Rev. Biophys. Biomol. Struct..

[92] MacKerell A.D., Bashford D., Bellott M., Dunbrack R.L., Evanseck J.D., Field M.J., Fischer S., Gao J., Guo H., Ha S. (1998). All-Atom Empirical Potential for Molecular Modeling and Dynamics Studies of Proteins. J. Phys. Chem. B.

[93] Jorgensen W.L., Chandrasekhar J., Madura J.D., Impey R.W., Klein M.L. (1983). Comparison of simple potential functions for simulating liquid water. J. Chem. Phys..

[94] Phillips J.C., Braun R., Wang W., Gumbart J., Tajkhorshid E., Villa E., Chipot C., Skeel R.D., Kalé L., Schulten K. (2005). Scalable Molecular Dynamics with NAMD. J. Comput. Chem..

[95] Di Pierro M., Elber R., Leimkuhler B. (2015). A Stochastic Algorithm for the Isobaric-Isothermal Ensemble with Ewald Summations for All Long Range Forces. J. Chem. Theory Comput..

[96] Martyna G.J., Klein M.L., Tuckerman M. (1992). Nosé–Hoover chains: The canonical ensemble via continuous dynamics. J. Chem. Phys..

[97] Martyna G.J., Tobias D.J., Klein M.L. (1994). Constant pressure molecular dynamics algorithms. J. Chem. Phys..

[98] Guerois R., Nielsen J.E., Serrano L. (2002). Predicting Changes in the Stability of Proteins and Protein Complexes: A Study of More than 1000 Mutations. J. Mol. Biol..

[99] Schymkowitz J., Borg J., Stricher F., Nys R., Rousseau F., Serrano L. (2005). The FoldX Web Server: An Online Force Field. Nucleic Acids Res..

[100] Van Durme J., Delgado J., Stricher F., Serrano L., Schymkowitz J., Rousseau F. (2011). A Graphical Interface for the FoldX Force Field. Bioinformatics.

[101] Christensen N.J., Kepp K.P. (2012). Accurate Stabilities of Laccase Mutants Predicted With a Modified FoldX Protocol. J. Chem. Inf. Model..

[102] Christensen N.J., Kepp K.P. (2013). Stability Mechanisms of Laccase Isoforms Using a Modified FoldX Protocol Applicable to Widely Different Proteins. J. Chem. Theory Comput..

[103] Dehouck Y., Kwasigroch J.M., Rooman M., Gilis D. (2013). BeAtMuSiC: Prediction of changes in protein-protein binding affinity on mutations. Nucleic Acids Res..

[104] Dehouck Y., Gilis D., Rooman M. (2006). A new generation of statistical potentials for proteins. Biophys. J..

[105] Dehouck Y., Grosfils A., Folch B., Gilis D., Bogaerts P., Rooman M. (2009). Fast and accurate predictions of protein stability changes upon mutations using statistical potentials and neural networks: PoPMuSiC-2.0. Bioinformatics.

