# Supplementary material for: Coevolution, Dynamics and Allostery Conspire in Shaping Cooperative Binding and Signal Transmission of the SARS-CoV-2 Spike Protein with Human Angiotensin-Converting Enzyme 2
Source: Int J Mol Sci. 2020 Nov 4;21(21):8268. doi: 10.3390/ijms21218268 (PMC7672574; doi:10.3390/ijms21218268)
Supplement: Supplementary file 1 [file ijms-21-08268-s001.zip › SUPPLEMENTARY_INFORMATION/IJMS_Supplementary_Information.docx]

**Supporting Information**

Coevolution, Dynamics and Allostery Conspire in Shaping Cooperative Binding and Signal Transmission of the SARS-CoV-2 Spike Protein with Human Angiotensin-Converting Enzyme 2

Gennady Verkhivker^1,2^*

^1^ Graduate Program in Computational and Data Sciences, Schmid College of Science and Technology, Chapman University, Orange, CA 92866, United States of America

^2^ Department of Biomedical and Pharmaceutical Sciences, Chapman University School of Pharmacy, Irvine, CA 92618, United States of America

***** Correspondence: verkhivk@chapman.edu; Tel.: +1-714-516-4586 (G.V)

##
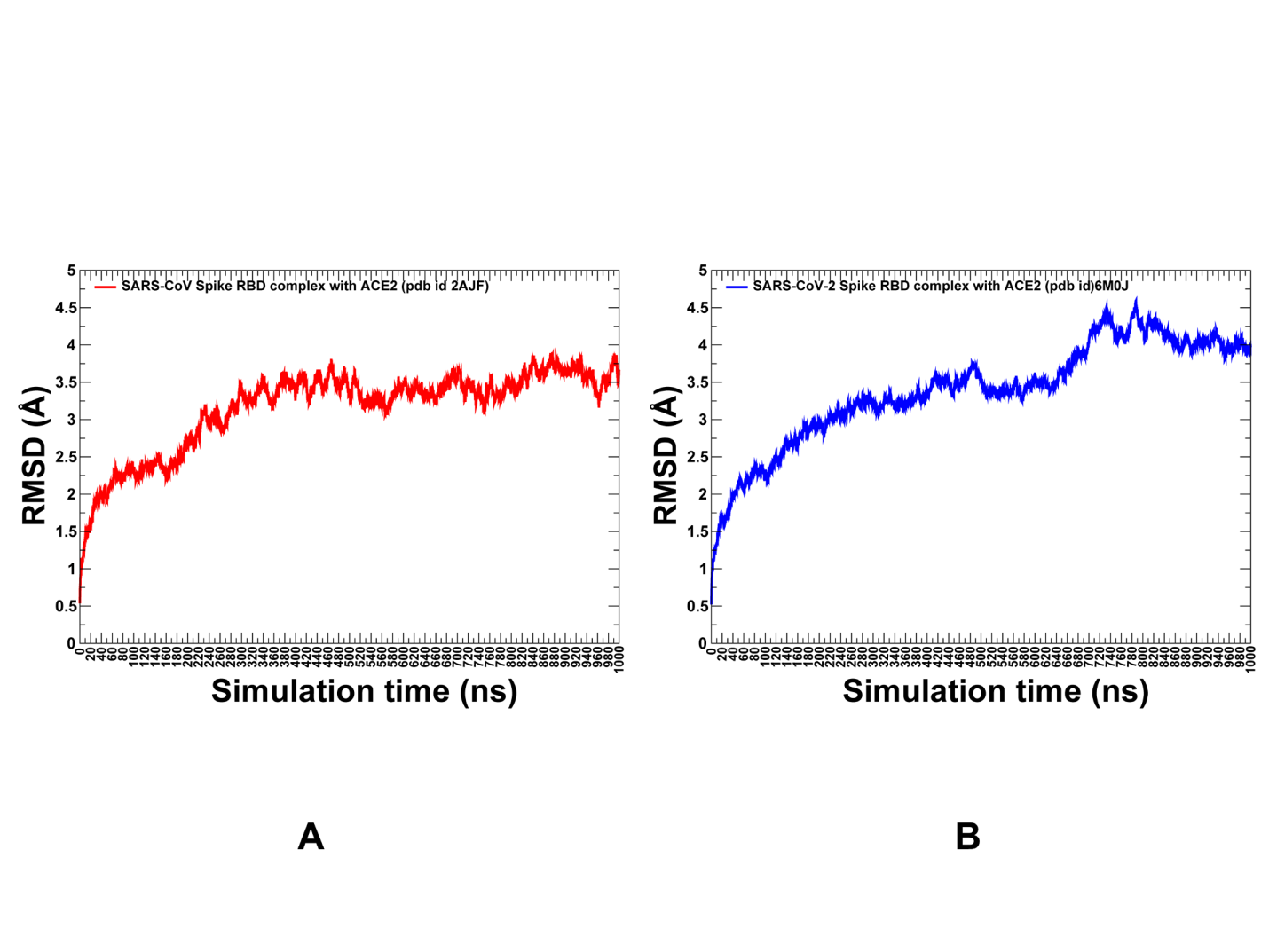


**Figure S1.** The root mean square deviation (RMSD) of the protein Cα atoms with respect to the initial structures of SARS-CoV/SARS-CoV-2 RBD complexes with ACE2 enzyme. (A) The RMSD values

of the all protein Cα atoms (shown in red line) obtained from all-atom MD simulations of the SARS-CoV Spike RBD complex with ACE2 (pdb id 2AJF). (B) The RMSD values of the all protein Cα atoms (shown in blue line) obtained from all-atom MD simulations of the SARS-CoV-2 Spike RBD complex with ACE2 (pdb id 6M0J).


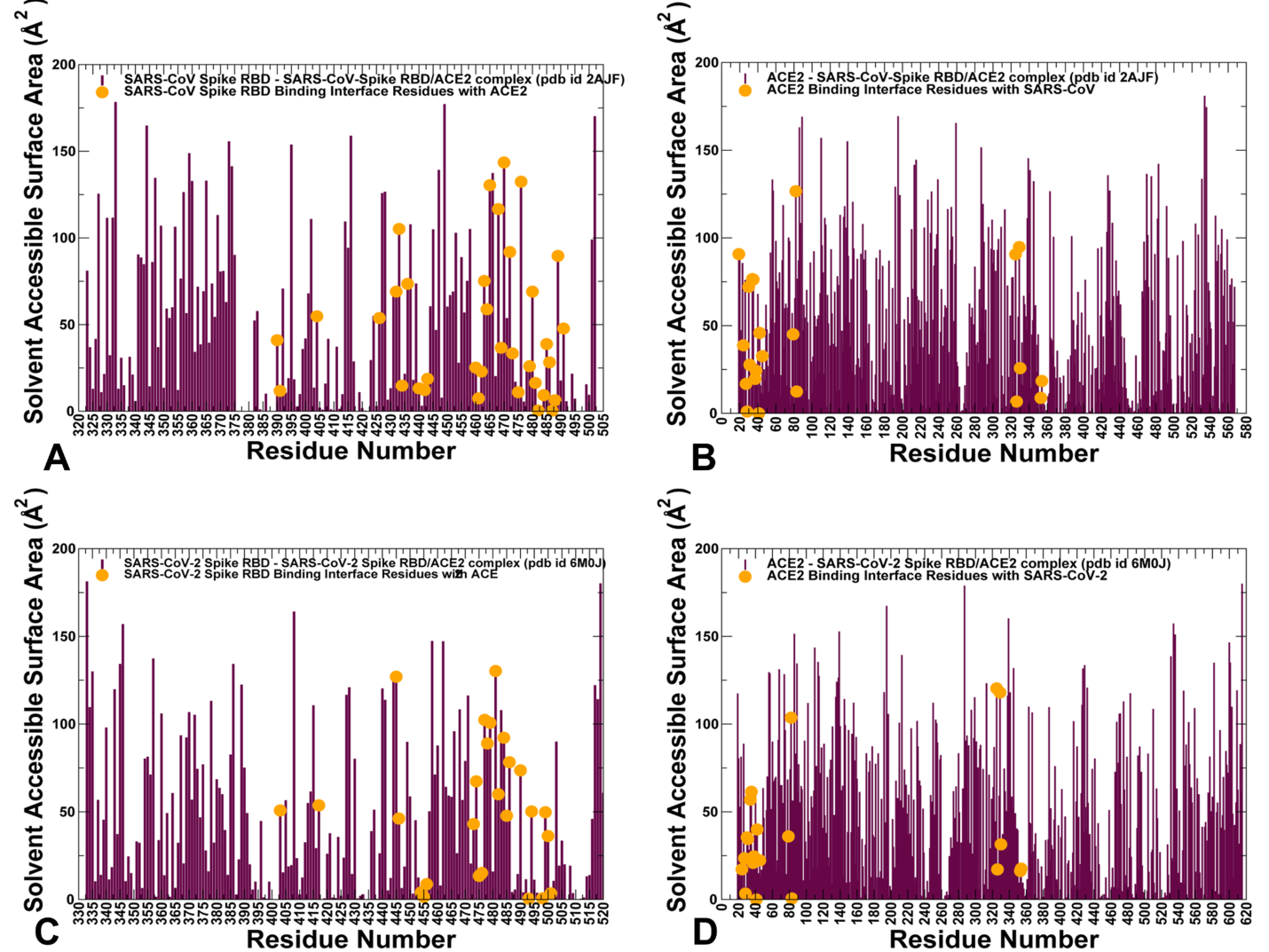


**Figure S2.** The residue solvent-accessible surface (SASA) profiles in SARS-CoV-RBD and SARS-CoV-2-RBD complexes with ACE2. (A) The SASA profile for SARS-CoV-RBD in the SARS-CoV-RBD complex with ACE2 (pdb id 2AJF). The binding interface residues that make contacts with ACE2 are highlighted in orange filled circles. (B) SASA profile for ACE2 residues in the SARS-CoV-RBD complex with ACE2 (pdb id 2AJF). The binding interface residues that make contacts with SARS-CoV-RBD are highlighted in orange filled circles. (C) The SASA profile for SARS-CoV-2-RBD protein in the SARS-CoV-2-RBD complex with ACE2 (pdb id 6M0J). (D) The SASA profile for ACE2 in the SARS-CoV-2-RBD complex with ACE2 (pdb id 6M0J).

**
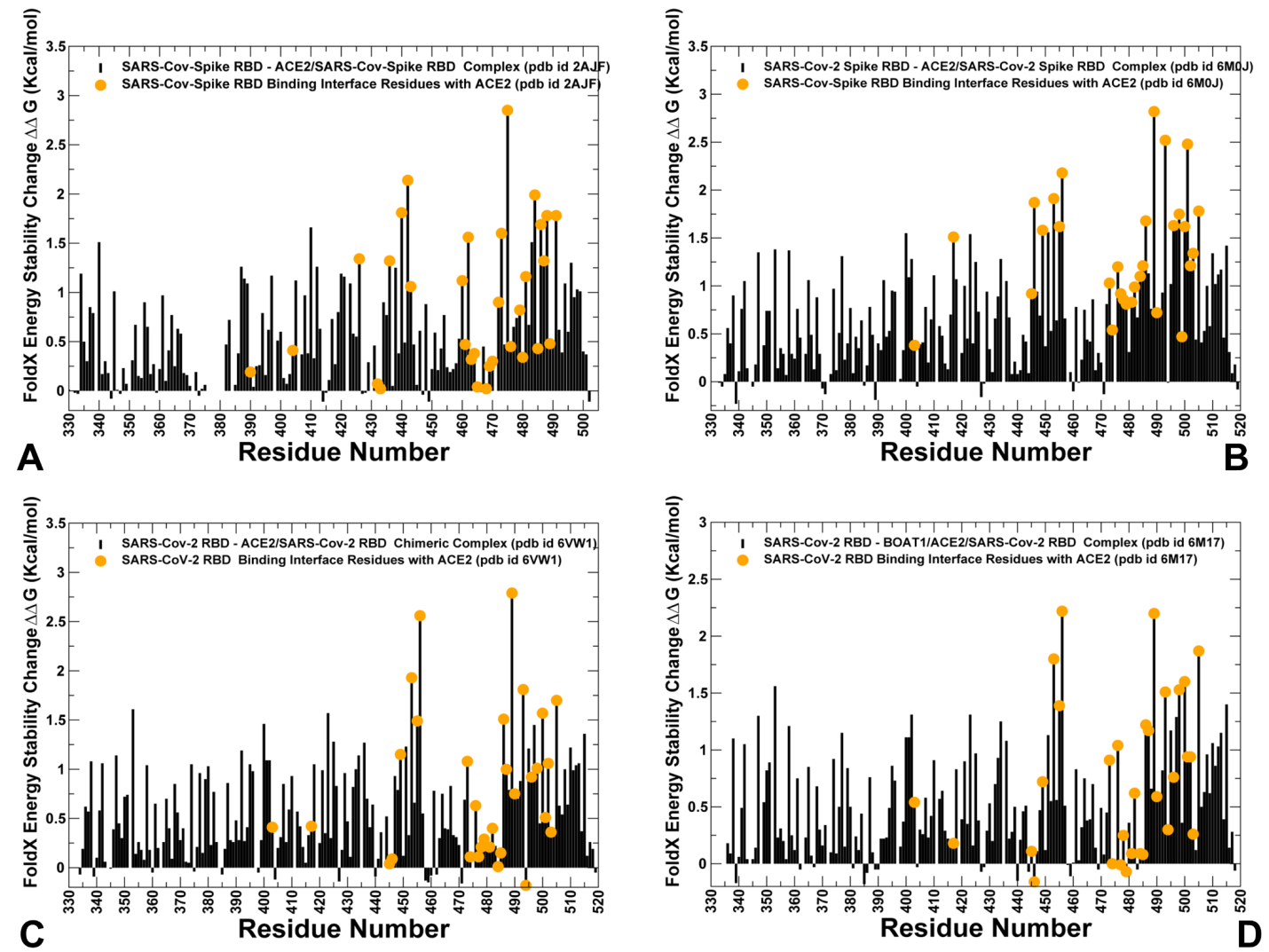
Figure S3.** FoldX protein stability analysis with a systematic alanine scanning of protein residues in the SARS-CoV and SARS-CoV-2 complexes with ACE2. (A) The FoldX protein stability changes upon alanine mutations in the SARS-CoV-RBD complex with ACE2 (pdb id 2AJF). The binding interface residues are highlighted in orange filled circles. The FoldX protein stability changes upon alanine mutations in the SARS-Cov-2-RBD complexes with ACE2 (pdb id 6M0J, panel B; pdb id 6VW1, panel C; and pdb id 6M17, panel D). The binding interface residues are determined for each complex based on the average interaction contacts that persist during MD simulation of a given complex.

**Table S1. The list of residues in the RBM region that differ between SARS-CoV-2 RBD and SARS-CoV-RBD.**

| **SARS-CoV-2** | **Residue Number** | **SARS-CoV** | **Residue Number** |
| --- | --- | --- | --- |
| ARG | 403 | LYS | 390 |
| LYS | 417 | VAL | 404 |
| ASN | 439 | ARG | 426 |
| SER | 443 | ALA | 430 |
| LYS | 444 | THR | 431 |
| VAL | 445 | SER | 432 |
| GLY | 446 | THR | 433 |
| LEU | 455 | TYR | 442 |
| PHE | 456 | LEU | 443 |
| LYS | 458 | HIS | 455 |
| SER | 459 | GLY | 446 |
| ASN | 460 | LYS | 447 |
| LYS | 462 | ARG | 449 |
| THR | 470 | ASN | 457 |
| GLU | 471 | VAL | 458 |
| ILE | 472 | PRO | 459 |
| TYR | 473 | PHE | 460 |
| GLN | 474 | SER | 461 |
| ALA | 475 | PRO | 462 |
| GLY | 476 | ASP | 463 |
| SER | 477 | GLY | 464 |
| THR | 478 | LYS | 465 |
| ASN | 481 | THR | 468 |
| GLY | 482 | PRO | 469 |
| GLU | 484 | PRO | 470 |
| GLY | 485 | ALA | 471 |
| PHE | 486 | LEU | 472 |
| PHE | 490 | TRP | 476 |
| GLN | 493 | ASN | 479 |
| SER | 494 | ASP | 480 |
| GLN | 498 | TYR | 484 |
| PRO | 499 | THR | 485 |
| ASN | 501 | THR | 487 |
| VAL | 503 | ILR | 489 |

**Table S2. The list of the interfacial contacts (ICs) in the SARS-CoV-RBD complex with ACE2 (pdb id 2AJF).**

| **SARS-CoV Residue** | **Number** | **ACE2 Residue** | **Number** |
| --- | --- | --- | --- |
| THR | 487 | LYS | 353 |
| THR | 486 | LEU | 45 |
| THR | 487 | ASN | 330 |
| TYR | 475 | GLN | 24 |
| ARG | 426 | GLU | 329 |
| TYR | 475 | THR | 27 |
| TYR | 491 | ARG | 393 |
| TYR | 484 | LEU | 45 |
| GLY | 482 | ASP | 38 |
| PRO | 462 | THR | 27 |
| TYR | 440 | HIS | 34 |
| TYR | 442 | LYS | 31 |
| TYR | 491 | LYS | 353 |
| THR | 487 | ASP | 355 |
| ASP | 463 | SER | 19 |
| PRO | 462 | GLN | 24 |
| PRO | 462 | SER | 19 |
| ASN | 473 | TYR | 83 |
| THR | 487 | GLY | 352 |
| TYR | 475 | LYS | 31 |
| GLY | 488 | GLY | 354 |
| ASN | 479 | HIS | 34 |
| LEU | 472 | MET | 82 |
| TYR | 436 | LYS | 353 |
| THR | 486 | ASP | 355 |
| ASN | 473 | GLN | 24 |
| TYR | 481 | LYS | 353 |
| TYR | 436 | GLN | 42 |
| TYR | 484 | ASP | 38 |
| TYR | 484 | TYR | 41 |
| ILE | 489 | GLN | 325 |
| PHE | 460 | THR | 27 |
| TYR | 475 | TYR | 83 |
| TYR | 442 | HIS | 34 |
| TYR | 484 | GLN | 42 |
| GLY | 488 | GLY | 352 |
| THR | 486 | ARG | 357 |
| GLY | 482 | LYS | 353 |
| TYR | 484 | LYS | 353 |
| ASN | 479 | ASP | 30 |
| ILE | 489 | GLY | 354 |
| TYR | 491 | GLU | 37 |
| THR | 487 | TYR | 41 |
| THR | 486 | GLY | 326 |
| LEU | 443 | THR | 27 |
| LEU | 472 | LEU | 79 |
| THR | 487 | GLY | 354 |
| THR | 486 | TYR | 41 |
| TYR | 442 | ASP | 30 |
| GLY | 488 | LYS | 353 |
| SER | 432 | LEU | 45 |
| THR | 486 | ASN | 330 |
| TYR | 491 | GLY | 354 |
| GLN | 492 | GLN | 325 |
| TYR | 436 | ASP | 38 |
| THR | 487 | GLY | 326 |
| GLY | 488 | ASP | 355 |
| TYR | 481 | ASP | 38 |
| THR | 485 | ASN | 330 |
| TYR | 475 | PHE | 28 |
| ARG | 426 | GLN | 325 |

**Table S3. The list of the interfacial contacts (ICs) in the SARS-CoV-2 RBD complex with ACE2 (pdb id 6M0J).**

| **SARS-CoV-2 Residue** | **Number** | **ACE2 Residue** | **Number** |
| --- | --- | --- | --- |
| GLN | 498 | LEU | 45 |
| TYR | 449 | GLN | 42 |
| TYR | 489 | GLN | 24 |
| GLN | 498 | LYS | 353 |
| GLN | 498 | TYR | 41 |
| TYR | 505 | GLY | 354 |
| PHE | 490 | LYS | 31 |
| TYR | 449 | ASP | 38 |
| SER | 477 | GLN | 24 |
| TYR | 489 | TYR | 83 |
| GLY | 496 | ASP | 38 |
| GLY | 502 | GLY | 354 |
| TYR | 505 | ARG | 393 |
| TYR | 505 | LYS | 353 |
| TYR | 453 | HIS | 34 |
| PHE | 486 | TYR | 83 |
| GLY | 502 | ASP | 355 |
| LEU | 455 | LYS | 31 |
| LYS | 417 | ASP | 30 |
| ASN | 501 | ASP | 355 |
| TYR | 489 | LYS | 31 |
| TYR | 489 | THR | 27 |
| GLN | 498 | GLN | 42 |
| ASN | 501 | TYR | 41 |
| GLN | 493 | GLU | 35 |
| ASN | 487 | GLN | 24 |
| GLN | 493 | LYS | 31 |
| PHE | 486 | GLN | 24 |
| GLY | 446 | GLN | 42 |
| ALA | 475 | SER | 19 |
| LEU | 455 | ASP | 30 |
| THR | 500 | GLY | 354 |
| GLY | 447 | GLN | 42 |
| PHE | 486 | MET | 82 |
| PHE | 486 | LEU | 79 |
| LYS | 417 | HIS | 34 |
| ASN | 501 | LYS | 353 |
| ASN | 487 | TYR | 83 |
| LEU | 455 | HIS | 34 |
| ALA | 475 | GLN | 24 |
| PHE | 497 | LYS | 353 |
| ALA | 475 | THR | 27 |
| TYR | 489 | PHE | 28 |
| PHE | 456 | ASP | 30 |
| ASN | 487 | PHE | 28 |
| THR | 500 | LEU | 45 |
| GLY | 496 | LYS | 353 |
| PHE | 456 | THR | 27 |
| GLU | 484 | LYS | 31 |
| GLY | 476 | GLN | 24 |
| GLN | 498 | ASP | 38 |
| THR | 500 | ASN | 330 |
| PHE | 456 | LYS | 31 |
| GLN | 493 | HIS | 34 |
| TYR | 505 | GLU | 37 |
| GLY | 446 | LEU | 45 |
| GLY | 502 | LYS | 353 |
| THR | 500 | TYR | 41 |
| TYR | 495 | LYS | 353 |
| TYR | 505 | ALA | 386 |
| THR | 500 | ASP | 355 |
| THR | 500 | LYS | 353 |
| VAL | 503 | GLY | 354 |
| ASN | 501 | GLY | 354 |
| TYR | 473 | THR | 27 |
| THR | 500 | ARG | 357 |

**Table S4. The ensemble-averaged contact time of the interfacial contacts (ICs) in the SARS-CoV-RBD complex with ACE2 (pdb id 2AJF).**

| **SARS-CoV Residue** | **Number** | **ACE2 Residue** | **Number** | **Contact time (%)** |
| --- | --- | --- | --- | --- |
| THR | 487 | LYS | 353 | 95.4 |
| THR | 486 | LEU | 45 | 88.3 |
| THR | 487 | ASN | 330 | 79.3 |
| TYR | 475 | GLN | 24 | 89.5 |
| ARG | 426 | GLU | 329 | 67.5 |
| TYR | 475 | THR | 27 | 80.3 |
| TYR | 491 | ARG | 393 | 79.6 |
| TYR | 484 | LEU | 45 | 78.6 |
| GLY | 482 | ASP | 38 | 69.3 |
| PRO | 462 | THR | 27 | 87.4 |
| TYR | 440 | HIS | 34 | 82.3 |
| TYR | 442 | LYS | 31 | 86.4 |
| TYR | 491 | LYS | 353 | 89.4 |
| THR | 487 | ASP | 355 | 86.4 |
| ASP | 463 | SER | 19 | 76.6 |
| PRO | 462 | GLN | 24 | 81.3 |
| PRO | 462 | SER | 19 | 82.5 |
| ASN | 473 | TYR | 83 | 86.3 |
| THR | 487 | GLY | 352 | 76.9 |
| TYR | 475 | LYS | 31 | 97.5 |
| GLY | 488 | GLY | 354 | 79.3 |
| ASN | 479 | HIS | 34 | 82.3 |
| LEU | 472 | MET | 82 | 80.3 |
| TYR | 436 | LYS | 353 | 88.6 |
| THR | 486 | ASP | 355 | 80.4 |
| ASN | 473 | GLN | 24 | 77.6 |
| TYR | 481 | LYS | 353 | 82.3 |
| TYR | 436 | GLN | 42 | 78.5 |
| TYR | 484 | ASP | 38 | 80.5 |
| TYR | 484 | TYR | 41 | 76.7 |
| ILE | 489 | GLN | 325 | 74.3 |
| PHE | 460 | THR | 27 | 69.5 |
| TYR | 475 | TYR | 83 | 78.5 |
| TYR | 442 | HIS | 34 | 80.3 |
| TYR | 484 | GLN | 42 | 82.3 |
| GLY | 488 | GLY | 352 | 75.4 |
| THR | 486 | ARG | 357 | 69.7 |
| GLY | 482 | LYS | 353 | 78.4 |
| TYR | 484 | LYS | 353 | 90.5 |
| ASN | 479 | ASP | 30 | 81.5 |
| ILE | 489 | GLY | 354 | 79.5 |
| TYR | 491 | GLU | 37 | 82.4 |
| THR | 487 | TYR | 41 | 88.5 |
| THR | 486 | GLY | 326 | 76.5 |
| LEU | 443 | THR | 27 | 86.9 |
| LEU | 472 | LEU | 79 | 75.6 |
| THR | 487 | GLY | 354 | 74.3 |
| THR | 486 | TYR | 41 | 83.4 |
| TYR | 442 | ASP | 30 | 80.6 |
| GLY | 488 | LYS | 353 | 88.3 |
| SER | 432 | LEU | 45 | 81.5 |
| THR | 486 | ASN | 330 | 79.6 |
| TYR | 491 | GLY | 354 | 83.6 |
| GLN | 492 | GLN | 325 | 69.7 |
| TYR | 436 | ASP | 38 | 89.7 |
| THR | 487 | GLY | 326 | 79.9 |
| GLY | 488 | ASP | 355 | 85.8 |
| TYR | 481 | ASP | 38 | 84.6 |
| THR | 485 | ASN | 330 | 82.9 |
| TYR | 475 | PHE | 28 | 70.1 |
| ARG | 426 | GLN | 325 | 79.6 |

**Table S5. The ensemble-averaged contact time of the interfacial contacts (ICs) in the SARS-CoV-2-RBD complex with ACE2 (pdb id 6M0J).**

| **SARS-CoV-2 Residue** | **Number** | **ACE2 Residue** | **Number** | **Contact time (%)** |
| --- | --- | --- | --- | --- |
| GLN | 498 | LEU | 45 | 93.5 |
| TYR | 449 | GLN | 42 | 90.3 |
| TYR | 489 | GLN | 24 | 91.2 |
| GLN | 498 | LYS | 353 | 92.3 |
| GLN | 498 | TYR | 41 | 94.5 |
| TYR | 505 | GLY | 354 | 89.5 |
| PHE | 490 | LYS | 31 | 93.2 |
| TYR | 449 | ASP | 38 | 94.6 |
| SER | 477 | GLN | 24 | 92.8 |
| TYR | 489 | TYR | 83 | 95.6 |
| GLY | 496 | ASP | 38 | 94.2 |
| GLY | 502 | GLY | 354 | 90.9 |
| TYR | 505 | ARG | 393 | 97.4 |
| TYR | 505 | LYS | 353 | 98.2 |
| TYR | 453 | HIS | 34 | 92.3 |
| PHE | 486 | TYR | 83 | 94.2 |
| GLY | 502 | ASP | 355 | 92.7 |
| LEU | 455 | LYS | 31 | 62.8 |
| LYS | 417 | ASP | 30 | 89.7 |
| ASN | 501 | ASP | 355 | 88.4 |
| TYR | 489 | LYS | 31 | 56.9 |
| TYR | 489 | THR | 27 | 89.9 |
| GLN | 498 | GLN | 42 | 90.4 |
| ASN | 501 | TYR | 41 | 95.6 |
| GLN | 493 | GLU | 35 | 87.6 |
| ASN | 487 | GLN | 24 | 84.8 |
| GLN | 493 | LYS | 31 | 94.3 |
| PHE | 486 | GLN | 24 | 90.2 |
| GLY | 446 | GLN | 42 | 89.5 |
| ALA | 475 | SER | 19 | 80.4 |
| LEU | 455 | ASP | 30 | 90.4 |
| THR | 500 | GLY | 354 | 87.6 |
| GLY | 447 | GLN | 42 | 85.9 |
| PHE | 486 | MET | 82 | 92.4 |
| PHE | 486 | LEU | 79 | 93.7 |
| LYS | 417 | HIS | 34 | 96.5 |
| ASN | 501 | LYS | 353 | 90.5 |
| ASN | 487 | TYR | 83 | 94.3 |
| LEU | 455 | HIS | 34 | 92.4 |
| ALA | 475 | GLN | 24 | 88.6 |
| PHE | 497 | LYS | 353 | 91.2 |
| ALA | 475 | THR | 27 | 92.3 |
| TYR | 489 | PHE | 28 | 86.3 |
| PHE | 456 | ASP | 30 | 92.4 |
| ASN | 487 | PHE | 28 | 94.3 |
| THR | 500 | LEU | 45 | 89.3 |
| GLY | 496 | LYS | 353 | 85.4 |
| PHE | 456 | THR | 27 | 88.2 |
| GLU | 484 | LYS | 31 | 90.4 |
| GLY | 476 | GLN | 24 | 86.7 |
| GLN | 498 | ASP | 38 | 92.5 |
| THR | 500 | ASN | 330 | 82.1 |
| PHE | 456 | LYS | 31 | 92.3 |
| GLN | 493 | HIS | 34 | 94.3 |
| TYR | 505 | GLU | 37 | 86.3 |
| GLY | 446 | LEU | 45 | 91.2 |
| GLY | 502 | LYS | 353 | 95.6 |
| THR | 500 | TYR | 41 | 96.8 |
| TYR | 495 | LYS | 353 | 90.4 |
| TYR | 505 | ALA | 386 | 84.3 |
| THR | 500 | ASP | 355 | 85.6 |
| THR | 500 | LYS | 353 | 93.3 |
| VAL | 503 | GLY | 354 | 88.6 |
| ASN | 501 | GLY | 354 | 90.4 |
| TYR | 473 | THR | 27 | 79.4 |
| THR | 500 | ARG | 357 | 81.2 |
