# Supplementary material for: Coevolution, Dynamics and Allostery Conspire in Shaping Cooperative Binding and Signal Transmission of the SARS-CoV-2 Spike Protein with Human Angiotensin-Converting Enzyme 2
Source: Int J Mol Sci. 2020 Nov 4;21(21):8268. doi: 10.3390/ijms21218268 (PMC7672574; doi:10.3390/ijms21218268)
Supplement: Supplementary file 1 [file ijms-21-08268-s001.zip › SUPPLEMENTARY_INFORMATION/IJMS_Supplementary_Information.pdf]

Supporting Information

Coevolution, Dynamics and Allostery Conspire in Shaping Cooperative Binding and Signal Transmission of the SARS-CoV-2 Spike Protein with Human Angiotensin-Converting Enzyme 2

Gennady Verkhivker[1,2*]

[1] Graduate Program in Computational and Data Sciences, Schmid College of Science and Technology, Chapman University, Orange, CA 92866, United States of America
[2] Department of Biomedical and Pharmaceutical Sciences, Chapman University School of Pharmacy, Irvine, CA 92618, United States of America
* Correspondence: verkhivk@chapman.edu; Tel.: +1-714-516-4586 (G.V)

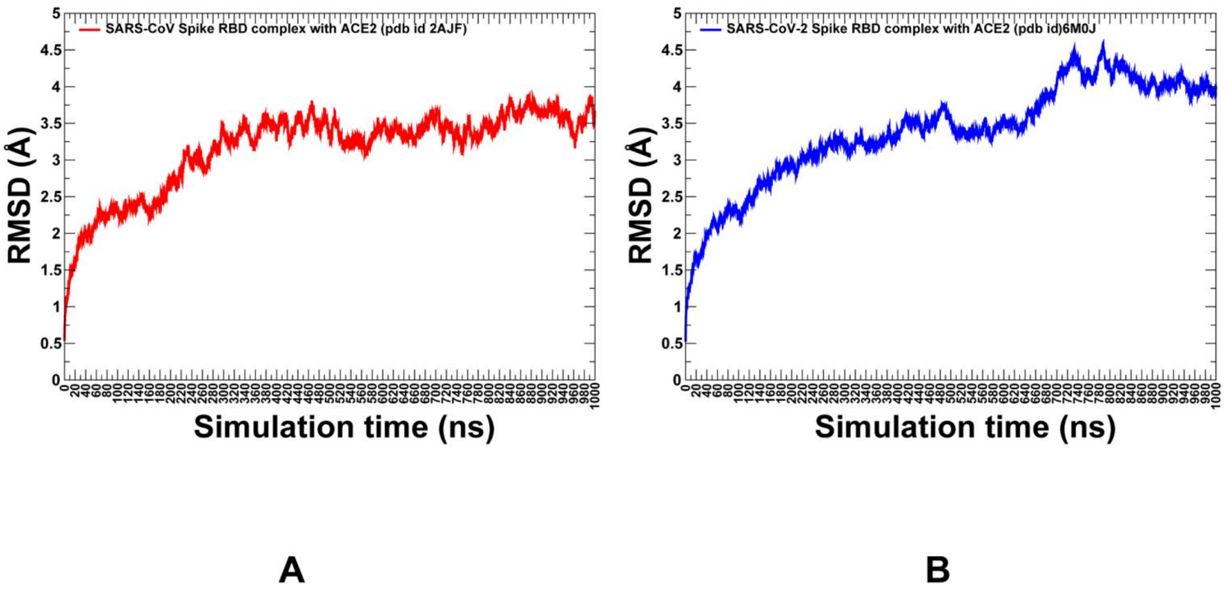

Figure S1. The root mean square deviation (RMSD) of the protein Cα atoms with respect to the initial structures of SARS-CoV/SARS-CoV-2 RBD complexes with ACE2 enzyme. (A) The RMSD values of the all protein Cα atoms (shown in red line) obtained from all-atom MD simulations of the SARS-CoV Spike RBD complex with ACE2 (pdb id 2AJF). (B) The RMSD values of the all protein Cα atoms (shown in blue line) obtained from all-atom MD simulations of the SARS-CoV-2 Spike RBD complex with ACE2 (pdb id 6M0J).

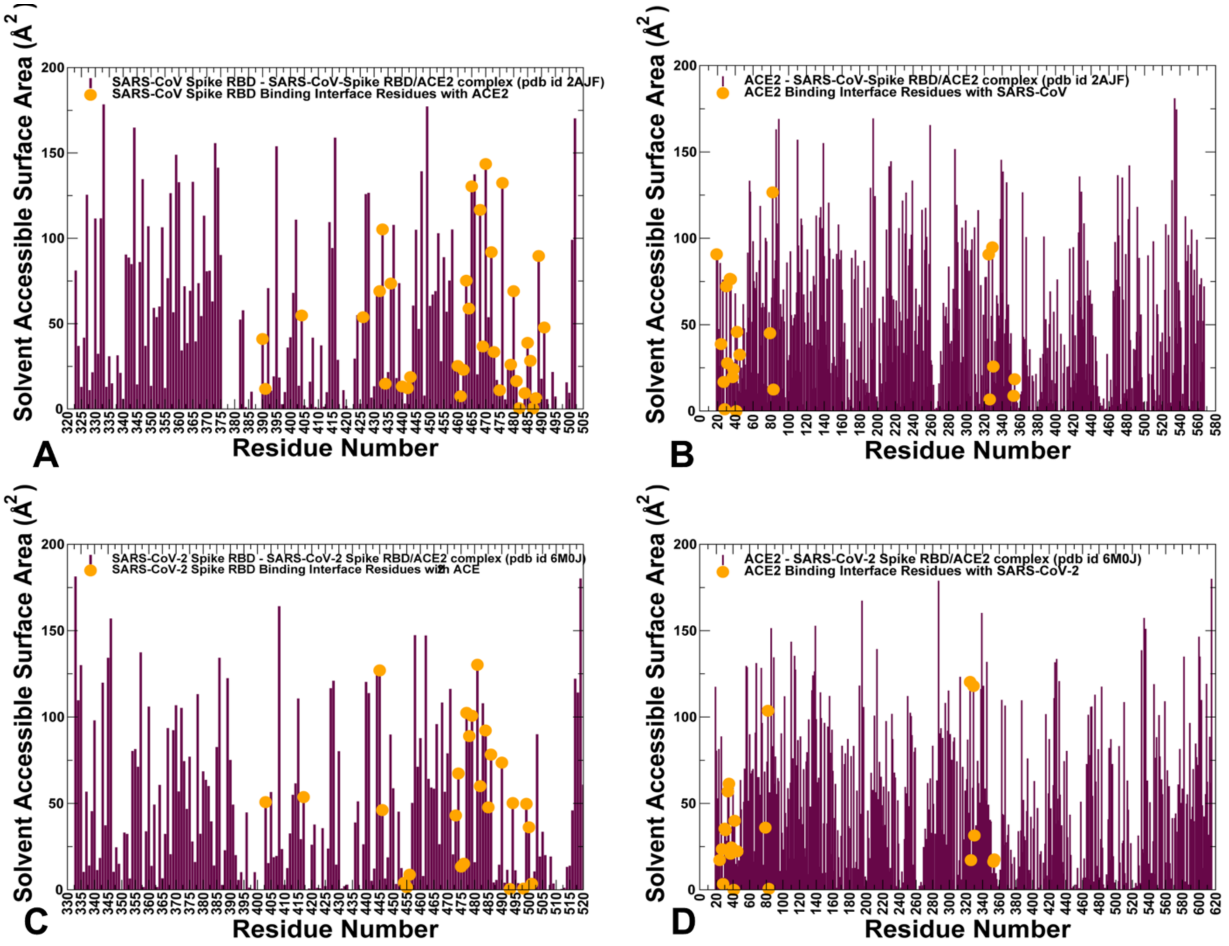

Figure S2. The residue solvent-accessible surface (SASA) profiles in SARS-CoV-RBD and SARS-CoV-2-RBD complexes with ACE2. (A) The SASA profile for SARS-CoV-RBD in the SARS-CoV-RBD complex with ACE2 (pdb id 2AJF). The binding interface residues that make contacts with ACE2 are highlighted in orange filled circles. (B) SASA profile for ACE2 residues in the SARS-CoV-RBD complex with ACE2 (pdb id 2AJF). The binding interface residues that make contacts with SARS-CoV-RBD are highlighted in orange filled circles. (C) The SASA profile for SARS-CoV-2-RBD protein in the SARS-CoV-2-RBD complex with ACE2 (pdb id 6M0J). (D) The SASA profile for ACE2 in the SARS-CoV-2-RBD complex with ACE2 (pdb id 6M0J).

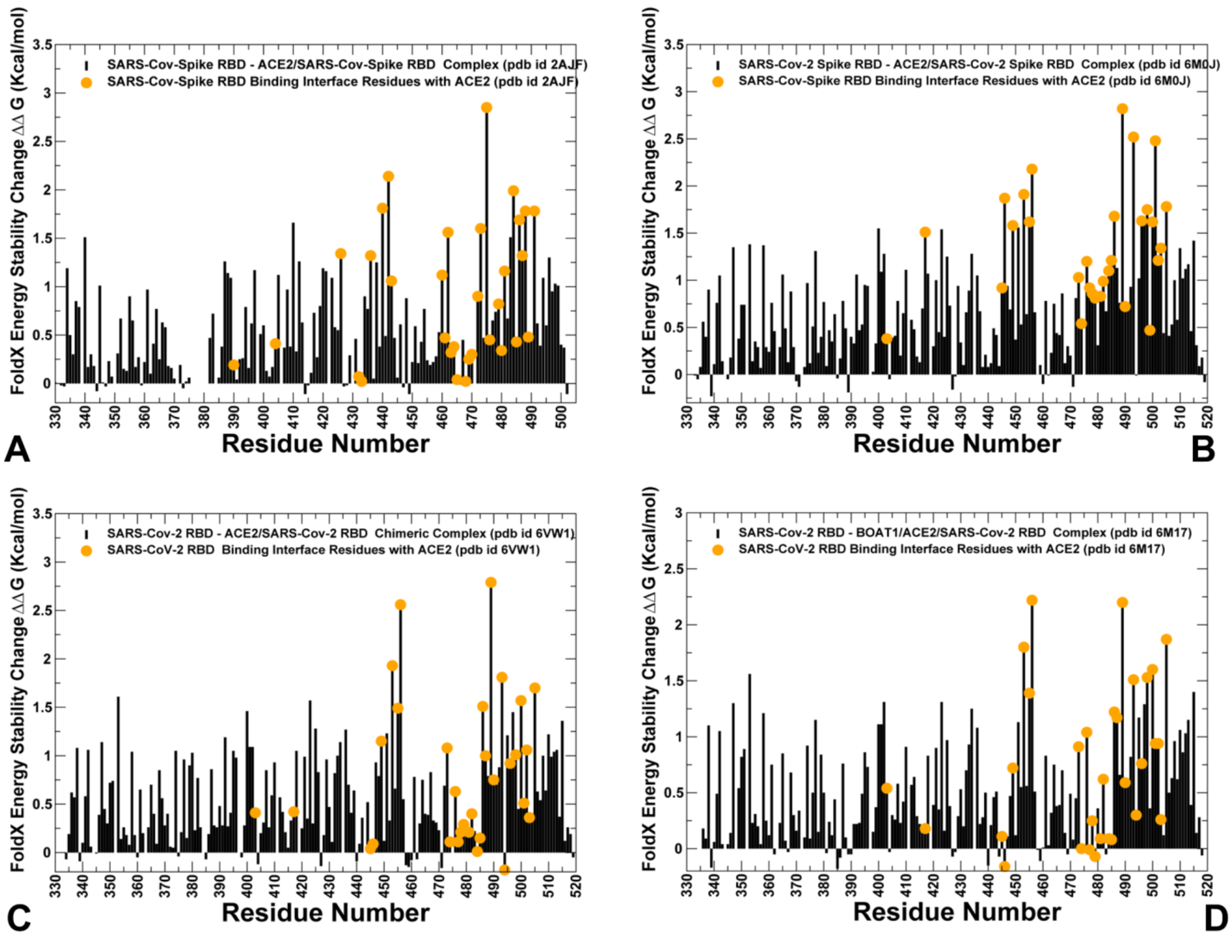

Figure S3. FoldX protein stability analysis with a systematic alanine scanning of protein residues in the SARS-CoV and SARS-CoV-2 complexes with ACE2. (A) The FoldX protein stability changes upon alanine mutations in the SARS-CoV-RBD complex with ACE2 (pdb id 2AJF). The binding interface residues are highlighted in orange filled circles. The FoldX protein stability changes upon alanine mutations in the SARS-Cov-2-RBD complexes with ACE2 (pdb id 6M0J, panel B; pdb id 6VW1, panel C; and pdb id 6M17, panel D). The binding interface residues are determined for each complex based on the average interaction contacts that persist during MD simulation of a given complex.

Table S1. The list of residues in the RBM region that differ between SARS-CoV-2 RBD and SARS-CoV-RBD.

SARS-CoV-2	Residue Number	SARS-CoV	Residue Number
ARG	403	LYS	390
LYS	417	VAL	404
ASN	439	ARG	426
SER	443	ALA	430
LYS	444	THR	431
VAL	445	SER	432
GLY	446	THR	433
LEU	455	TYR	442
PHE	456	LEU	443
LYS	458	HIS	455
SER	459	GLY	446
ASN	460	LYS	447
LYS	462	ARG	449
THR	470	ASN	457
GLU	471	VAL	458
ILE	472	PRO	459
TYR	473	PHE	460
GLN	474	SER	461
ALA	475	PRO	462
GLY	476	ASP	463
SER	477	GLY	464
THR	478	LYS	465
ASN	481	THR	468
GLY	482	PRO	469
GLU	484	PRO	470
GLY	485	ALA	471
PHE	486	LEU	472
PHE	490	TRP	476
GLN	493	ASN	479
SER	494	ASP	480
GLN	498	TYR	484
PRO	499	THR	485
ASN	501	THR	487
VAL	503	ILR	489

Table S2. The list of the interfacial contacts (ICs) in the SARS-CoV-RBD complex with ACE2 (pdb id 2AJF).

SARS-CoV Residue	Num ber	ACE2 Residue	Numb er
THR	487	LYS	353
THR	486	LEU	45
THR	487	ASN	330
TYR	475	GLN	24
ARG	426	GLU	329
TYR	475	THR	27
TYR	491	ARG	393
TYR	484	LEU	45
GLY	482	ASP	38
PRO	462	THR	27
TYR	440	HIS	34
TYR	442	LYS	31
TYR	491	LYS	353
THR	487	ASP	355
ASP	463	SER	19
PRO	462	GLN	24
PRO	462	SER	19
ASN	473	TYR	83
THR	487	GLY	352
TYR	475	LYS	31
GLY	488	GLY	354
ASN	479	HIS	34
LEU	472	MET	82
TYR	436	LYS	353
THR	486	ASP	355
ASN	473	GLN	24
TYR	481	LYS	353
TYR	436	GLN	42
TYR	484	ASP	38
TYR	484	TYR	41
ILE	489	GLN	325
PHE	460	THR	27
TYR	475	TYR	83
TYR	442	HIS	34
TYR	484	GLN	42
GLY	488	GLY	352
THR	486	ARG	357

GLY	482	LYS	353
TYR	484	LYS	353
ASN	479	ASP	30
ILE	489	GLY	354
TYR	491	GLU	37
THR	487	TYR	41
THR	486	GLY	326
LEU	443	THR	27
LEU	472	LEU	79
THR	487	GLY	354
THR	486	TYR	41
TYR	442	ASP	30
GLY	488	LYS	353
SER	432	LEU	45
THR	486	ASN	330
TYR	491	GLY	354
GLN	492	GLN	325
TYR	436	ASP	38
THR	487	GLY	326
GLY	488	ASP	355
TYR	481	ASP	38
THR	485	ASN	330
TYR	475	PHE	28
ARG	426	GLN	325

Table S3. The list of the interfacial contacts (ICs) in the SARS-CoV-2 RBD complex with ACE2 (pdb id 6M0J).

SARS-CoV-2 Residue	Num ber	ACE2 Residue	Num ber
GLN	498	LEU	45
TYR	449	GLN	42
TYR	489	GLN	24
GLN	498	LYS	353
GLN	498	TYR	41
TYR	505	GLY	354
PHE	490	LYS	31
TYR	449	ASP	38
SER	477	GLN	24
TYR	489	TYR	83
GLY	496	ASP	38
GLY	502	GLY	354
TYR	505	ARG	393
TYR	505	LYS	353
TYR	453	HIS	34
PHE	486	TYR	83
GLY	502	ASP	355
LEU	455	LYS	31
LYS	417	ASP	30
ASN	501	ASP	355
TYR	489	LYS	31
TYR	489	THR	27
GLN	498	GLN	42
ASN	501	TYR	41
GLN	493	GLU	35
ASN	487	GLN	24
GLN	493	LYS	31
PHE	486	GLN	24
GLY	446	GLN	42
ALA	475	SER	19
LEU	455	ASP	30
THR	500	GLY	354
GLY	447	GLN	42
PHE	486	MET	82
PHE	486	LEU	79
LYS	417	HIS	34
ASN	501	LYS	353

ASN	487	TYR	83
LEU	455	HIS	34
ALA	475	GLN	24
PHE	497	LYS	353
ALA	475	THR	27
TYR	489	PHE	28
PHE	456	ASP	30
ASN	487	PHE	28
THR	500	LEU	45
GLY	496	LYS	353
PHE	456	THR	27
GLU	484	LYS	31
GLY	476	GLN	24
GLN	498	ASP	38
THR	500	ASN	330
PHE	456	LYS	31
GLN	493	HIS	34
TYR	505	GLU	37
GLY	446	LEU	45
GLY	502	LYS	353
THR	500	TYR	41
TYR	495	LYS	353
TYR	505	ALA	386
THR	500	ASP	355
THR	500	LYS	353
VAL	503	GLY	354
ASN	501	GLY	354
TYR	473	THR	27
THR	500	ARG	357

Table S4. The ensemble-averaged contact time of the interfacial contacts (ICs) in the SARS-CoV-RBD complex with ACE2 (pdb id 2AJF).

SARS-CoV Residue	Number	ACE2 Residue	Number	Contact time (%)
THR	487	LYS	353	95.4
THR	486	LEU	45	88.3
THR	487	ASN	330	79.3
TYR	475	GLN	24	89.5
ARG	426	GLU	329	67.5
TYR	475	THR	27	80.3
TYR	491	ARG	393	79.6
TYR	484	LEU	45	78.6
GLY	482	ASP	38	69.3
PRO	462	THR	27	87.4
TYR	440	HIS	34	82.3
TYR	442	LYS	31	86.4
TYR	491	LYS	353	89.4
THR	487	ASP	355	86.4
ASP	463	SER	19	76.6
PRO	462	GLN	24	81.3
PRO	462	SER	19	82.5
ASN	473	TYR	83	86.3
THR	487	GLY	352	76.9
TYR	475	LYS	31	97.5
GLY	488	GLY	354	79.3
ASN	479	HIS	34	82.3
LEU	472	MET	82	80.3
TYR	436	LYS	353	88.6
THR	486	ASP	355	80.4
ASN	473	GLN	24	77.6
TYR	481	LYS	353	82.3
TYR	436	GLN	42	78.5
TYR	484	ASP	38	80.5
TYR	484	TYR	41	76.7
ILE	489	GLN	325	74.3
PHE	460	THR	27	69.5
TYR	475	TYR	83	78.5
TYR	442	HIS	34	80.3
TYR	484	GLN	42	82.3
GLY	488	GLY	352	75.4
THR	486	ARG	357	69.7

GLY	482	LYS	353	78.4
TYR	484	LYS	353	90.5
ASN	479	ASP	30	81.5
ILE	489	GLY	354	79.5
TYR	491	GLU	37	82.4
THR	487	TYR	41	88.5
THR	486	GLY	326	76.5
LEU	443	THR	27	86.9
LEU	472	LEU	79	75.6
THR	487	GLY	354	74.3
THR	486	TYR	41	83.4
TYR	442	ASP	30	80.6
GLY	488	LYS	353	88.3
SER	432	LEU	45	81.5
THR	486	ASN	330	79.6
TYR	491	GLY	354	83.6
GLN	492	GLN	325	69.7
TYR	436	ASP	38	89.7
THR	487	GLY	326	79.9
GLY	488	ASP	355	85.8
TYR	481	ASP	38	84.6
THR	485	ASN	330	82.9
TYR	475	PHE	28	70.1
ARG	426	GLN	325	79.6

Table S5. The ensemble-averaged contact time of the interfacial contacts (ICs) in the SARS-CoV-2-RBD complex with ACE2 (pdb id 6M0J).

SARS-CoV-2 Residue	Number	ACE2 Residue	Number	Contact time (%)
GLN	498	LEU	45	93.5
TYR	449	GLN	42	90.3
TYR	489	GLN	24	91.2
GLN	498	LYS	353	92.3
GLN	498	TYR	41	94.5
TYR	505	GLY	354	89.5
PHE	490	LYS	31	93.2
TYR	449	ASP	38	94.6
SER	477	GLN	24	92.8
TYR	489	TYR	83	95.6
GLY	496	ASP	38	94.2
GLY	502	GLY	354	90.9
TYR	505	ARG	393	97.4
TYR	505	LYS	353	98.2
TYR	453	HIS	34	92.3
PHE	486	TYR	83	94.2
GLY	502	ASP	355	92.7
LEU	455	LYS	31	62.8
LYS	417	ASP	30	89.7
ASN	501	ASP	355	88.4
TYR	489	LYS	31	56.9
TYR	489	THR	27	89.9
GLN	498	GLN	42	90.4
ASN	501	TYR	41	95.6
GLN	493	GLU	35	87.6
ASN	487	GLN	24	84.8
GLN	493	LYS	31	94.3
PHE	486	GLN	24	90.2
GLY	446	GLN	42	89.5
ALA	475	SER	19	80.4
LEU	455	ASP	30	90.4
THR	500	GLY	354	87.6
GLY	447	GLN	42	85.9
PHE	486	MET	82	92.4
PHE	486	LEU	79	93.7
LYS	417	HIS	34	96.5

ASN	501	LYS	353	90.5
ASN	487	TYR	83	94.3
LEU	455	HIS	34	92.4
ALA	475	GLN	24	88.6
PHE	497	LYS	353	91.2
ALA	475	THR	27	92.3
TYR	489	PHE	28	86.3
PHE	456	ASP	30	92.4
ASN	487	PHE	28	94.3
THR	500	LEU	45	89.3
GLY	496	LYS	353	85.4
PHE	456	THR	27	88.2
GLU	484	LYS	31	90.4
GLY	476	GLN	24	86.7
GLN	498	ASP	38	92.5
THR	500	ASN	330	82.1
PHE	456	LYS	31	92.3
GLN	493	HIS	34	94.3
TYR	505	GLU	37	86.3
GLY	446	LEU	45	91.2
GLY	502	LYS	353	95.6
THR	500	TYR	41	96.8
TYR	495	LYS	353	90.4
TYR	505	ALA	386	84.3
THR	500	ASP	355	85.6
THR	500	LYS	353	93.3
VAL	503	GLY	354	88.6
ASN	501	GLY	354	90.4
TYR	473	THR	27	79.4
THR	500	ARG	357	81.2
