# Supplementary material for: Coevolution, Dynamics and Allostery Conspire in Shaping Cooperative Binding and Signal Transmission of the SARS-CoV-2 Spike Protein with Human Angiotensin-Converting Enzyme 2
Source: Int J Mol Sci. 2020 Nov 4;21(21):8268. doi: 10.3390/ijms21218268 (PMC7672574; doi:10.3390/ijms21218268)
Supplement: Supplementary file 1 [file ijms-21-08268-s001.zip › SUPPLEMENTARY_INFORMATION/Table S1.docx]

**Table S1. The list of residues in the RBM region that differ between SARS-CoV-2 RBD and SARS-CoV-RBD.**

| **SARS-CoV-2** | **Residue Number** | **SARS-CoV** | **Residue Number** |
| --- | --- | --- | --- |
| ARG | 403 | LYS | 390 |
| LYS | 417 | VAL | 404 |
| ASN | 439 | ARG | 426 |
| SER | 443 | ALA | 430 |
| LYS | 444 | THR | 431 |
| VAL | 445 | SER | 432 |
| GLY | 446 | THR | 433 |
| LEU | 455 | TYR | 442 |
| PHE | 456 | LEU | 443 |
| LYS | 458 | HIS | 455 |
| SER | 459 | GLY | 446 |
| ASN | 460 | LYS | 447 |
| LYS | 462 | ARG | 449 |
| THR | 470 | ASN | 457 |
| GLU | 471 | VAL | 458 |
| ILE | 472 | PRO | 459 |
| TYR | 473 | PHE | 460 |
| GLN | 474 | SER | 461 |
| ALA | 475 | PRO | 462 |
| GLY | 476 | ASP | 463 |
| SER | 477 | GLY | 464 |
| THR | 478 | LYS | 465 |
| ASN | 481 | THR | 468 |
| GLY | 482 | PRO | 469 |
| GLU | 484 | PRO | 470 |
| GLY | 485 | ALA | 471 |
| PHE | 486 | LEU | 472 |
| PHE | 490 | TRP | 476 |
| GLN | 493 | ASN | 479 |
| SER | 494 | ASP | 480 |
| GLN | 498 | TYR | 484 |
| PRO | 499 | THR | 485 |
| ASN | 501 | THR | 487 |
| VAL | 503 | ILR | 489 |
