# Supplementary material for: Coevolution, Dynamics and Allostery Conspire in Shaping Cooperative Binding and Signal Transmission of the SARS-CoV-2 Spike Protein with Human Angiotensin-Converting Enzyme 2
Source: Int J Mol Sci. 2020 Nov 4;21(21):8268. doi: 10.3390/ijms21218268 (PMC7672574; doi:10.3390/ijms21218268)
Supplement: Supplementary file 1 [file ijms-21-08268-s001.zip › SUPPLEMENTARY_INFORMATION/Table S2.docx]

**Table S2. The list of the interfacial contacts (ICs) in the SARS-CoV-RBD complex with ACE2 (pdb id 2AJF).**

| **SARS-CoV Residue** | **Number** | **ACE2 Residue** | **Number** |
| --- | --- | --- | --- |
| THR | 487 | LYS | 353 |
| THR | 486 | LEU | 45 |
| THR | 487 | ASN | 330 |
| TYR | 475 | GLN | 24 |
| ARG | 426 | GLU | 329 |
| TYR | 475 | THR | 27 |
| TYR | 491 | ARG | 393 |
| TYR | 484 | LEU | 45 |
| GLY | 482 | ASP | 38 |
| PRO | 462 | THR | 27 |
| TYR | 440 | HIS | 34 |
| TYR | 442 | LYS | 31 |
| TYR | 491 | LYS | 353 |
| THR | 487 | ASP | 355 |
| ASP | 463 | SER | 19 |
| PRO | 462 | GLN | 24 |
| PRO | 462 | SER | 19 |
| ASN | 473 | TYR | 83 |
| THR | 487 | GLY | 352 |
| TYR | 475 | LYS | 31 |
| GLY | 488 | GLY | 354 |
| ASN | 479 | HIS | 34 |
| LEU | 472 | MET | 82 |
| TYR | 436 | LYS | 353 |
| THR | 486 | ASP | 355 |
| ASN | 473 | GLN | 24 |
| TYR | 481 | LYS | 353 |
| TYR | 436 | GLN | 42 |
| TYR | 484 | ASP | 38 |
| TYR | 484 | TYR | 41 |
| ILE | 489 | GLN | 325 |
| PHE | 460 | THR | 27 |
| TYR | 475 | TYR | 83 |
| TYR | 442 | HIS | 34 |
| TYR | 484 | GLN | 42 |
| GLY | 488 | GLY | 352 |
| THR | 486 | ARG | 357 |
| GLY | 482 | LYS | 353 |
| TYR | 484 | LYS | 353 |
| ASN | 479 | ASP | 30 |
| ILE | 489 | GLY | 354 |
| TYR | 491 | GLU | 37 |
| THR | 487 | TYR | 41 |
| THR | 486 | GLY | 326 |
| LEU | 443 | THR | 27 |
| LEU | 472 | LEU | 79 |
| THR | 487 | GLY | 354 |
| THR | 486 | TYR | 41 |
| TYR | 442 | ASP | 30 |
| GLY | 488 | LYS | 353 |
| SER | 432 | LEU | 45 |
| THR | 486 | ASN | 330 |
| TYR | 491 | GLY | 354 |
| GLN | 492 | GLN | 325 |
| TYR | 436 | ASP | 38 |
| THR | 487 | GLY | 326 |
| GLY | 488 | ASP | 355 |
| TYR | 481 | ASP | 38 |
| THR | 485 | ASN | 330 |
| TYR | 475 | PHE | 28 |
| ARG | 426 | GLN | 325 |
