# Supplementary material for: Coevolution, Dynamics and Allostery Conspire in Shaping Cooperative Binding and Signal Transmission of the SARS-CoV-2 Spike Protein with Human Angiotensin-Converting Enzyme 2
Source: Int J Mol Sci. 2020 Nov 4;21(21):8268. doi: 10.3390/ijms21218268 (PMC7672574; doi:10.3390/ijms21218268)
Supplement: Supplementary file 1 [file ijms-21-08268-s001.zip › SUPPLEMENTARY_INFORMATION/Table S3.docx]

**Table S3. The list of the interfacial contacts (ICs) in the SARS-CoV-2 RBD complex with ACE2 (pdb id 6M0J).**

| **SARS-CoV-2 Residue** | **Number** | **ACE2 Residue** | **Number** |
| --- | --- | --- | --- |
| GLN | 498 | LEU | 45 |
| TYR | 449 | GLN | 42 |
| TYR | 489 | GLN | 24 |
| GLN | 498 | LYS | 353 |
| GLN | 498 | TYR | 41 |
| TYR | 505 | GLY | 354 |
| PHE | 490 | LYS | 31 |
| TYR | 449 | ASP | 38 |
| SER | 477 | GLN | 24 |
| TYR | 489 | TYR | 83 |
| GLY | 496 | ASP | 38 |
| GLY | 502 | GLY | 354 |
| TYR | 505 | ARG | 393 |
| TYR | 505 | LYS | 353 |
| TYR | 453 | HIS | 34 |
| PHE | 486 | TYR | 83 |
| GLY | 502 | ASP | 355 |
| LEU | 455 | LYS | 31 |
| LYS | 417 | ASP | 30 |
| ASN | 501 | ASP | 355 |
| TYR | 489 | LYS | 31 |
| TYR | 489 | THR | 27 |
| GLN | 498 | GLN | 42 |
| ASN | 501 | TYR | 41 |
| GLN | 493 | GLU | 35 |
| ASN | 487 | GLN | 24 |
| GLN | 493 | LYS | 31 |
| PHE | 486 | GLN | 24 |
| GLY | 446 | GLN | 42 |
| ALA | 475 | SER | 19 |
| LEU | 455 | ASP | 30 |
| THR | 500 | GLY | 354 |
| GLY | 447 | GLN | 42 |
| PHE | 486 | MET | 82 |
| PHE | 486 | LEU | 79 |
| LYS | 417 | HIS | 34 |
| ASN | 501 | LYS | 353 |
| ASN | 487 | TYR | 83 |
| LEU | 455 | HIS | 34 |
| ALA | 475 | GLN | 24 |
| PHE | 497 | LYS | 353 |
| ALA | 475 | THR | 27 |
| TYR | 489 | PHE | 28 |
| PHE | 456 | ASP | 30 |
| ASN | 487 | PHE | 28 |
| THR | 500 | LEU | 45 |
| GLY | 496 | LYS | 353 |
| PHE | 456 | THR | 27 |
| GLU | 484 | LYS | 31 |
| GLY | 476 | GLN | 24 |
| GLN | 498 | ASP | 38 |
| THR | 500 | ASN | 330 |
| PHE | 456 | LYS | 31 |
| GLN | 493 | HIS | 34 |
| TYR | 505 | GLU | 37 |
| GLY | 446 | LEU | 45 |
| GLY | 502 | LYS | 353 |
| THR | 500 | TYR | 41 |
| TYR | 495 | LYS | 353 |
| TYR | 505 | ALA | 386 |
| THR | 500 | ASP | 355 |
| THR | 500 | LYS | 353 |
| VAL | 503 | GLY | 354 |
| ASN | 501 | GLY | 354 |
| TYR | 473 | THR | 27 |
| THR | 500 | ARG | 357 |
