# Supplementary material for: Coevolution, Dynamics and Allostery Conspire in Shaping Cooperative Binding and Signal Transmission of the SARS-CoV-2 Spike Protein with Human Angiotensin-Converting Enzyme 2
Source: Int J Mol Sci. 2020 Nov 4;21(21):8268. doi: 10.3390/ijms21218268 (PMC7672574; doi:10.3390/ijms21218268)
Supplement: Supplementary file 1 [file ijms-21-08268-s001.zip › SUPPLEMENTARY_INFORMATION/Table S4.docx]

**Table S4. The ensemble-averaged contact time of the interfacial contacts (ICs) in the SARS-CoV-RBD complex with ACE2 (pdb id 2AJF).**

| **SARS-CoV Residue** | **Number** | **ACE2 Residue** | **Number** | **Contact time (%)** |
| --- | --- | --- | --- | --- |
| THR | 487 | LYS | 353 | 95.4 |
| THR | 486 | LEU | 45 | 88.3 |
| THR | 487 | ASN | 330 | 79.3 |
| TYR | 475 | GLN | 24 | 89.5 |
| ARG | 426 | GLU | 329 | 67.5 |
| TYR | 475 | THR | 27 | 80.3 |
| TYR | 491 | ARG | 393 | 79.6 |
| TYR | 484 | LEU | 45 | 78.6 |
| GLY | 482 | ASP | 38 | 69.3 |
| PRO | 462 | THR | 27 | 87.4 |
| TYR | 440 | HIS | 34 | 82.3 |
| TYR | 442 | LYS | 31 | 86.4 |
| TYR | 491 | LYS | 353 | 89.4 |
| THR | 487 | ASP | 355 | 86.4 |
| ASP | 463 | SER | 19 | 76.6 |
| PRO | 462 | GLN | 24 | 81.3 |
| PRO | 462 | SER | 19 | 82.5 |
| ASN | 473 | TYR | 83 | 86.3 |
| THR | 487 | GLY | 352 | 76.9 |
| TYR | 475 | LYS | 31 | 97.5 |
| GLY | 488 | GLY | 354 | 79.3 |
| ASN | 479 | HIS | 34 | 82.3 |
| LEU | 472 | MET | 82 | 80.3 |
| TYR | 436 | LYS | 353 | 88.6 |
| THR | 486 | ASP | 355 | 80.4 |
| ASN | 473 | GLN | 24 | 77.6 |
| TYR | 481 | LYS | 353 | 82.3 |
| TYR | 436 | GLN | 42 | 78.5 |
| TYR | 484 | ASP | 38 | 80.5 |
| TYR | 484 | TYR | 41 | 76.7 |
| ILE | 489 | GLN | 325 | 74.3 |
| PHE | 460 | THR | 27 | 69.5 |
| TYR | 475 | TYR | 83 | 78.5 |
| TYR | 442 | HIS | 34 | 80.3 |
| TYR | 484 | GLN | 42 | 82.3 |
| GLY | 488 | GLY | 352 | 75.4 |
| THR | 486 | ARG | 357 | 69.7 |
| GLY | 482 | LYS | 353 | 78.4 |
| TYR | 484 | LYS | 353 | 90.5 |
| ASN | 479 | ASP | 30 | 81.5 |
| ILE | 489 | GLY | 354 | 79.5 |
| TYR | 491 | GLU | 37 | 82.4 |
| THR | 487 | TYR | 41 | 88.5 |
| THR | 486 | GLY | 326 | 76.5 |
| LEU | 443 | THR | 27 | 86.9 |
| LEU | 472 | LEU | 79 | 75.6 |
| THR | 487 | GLY | 354 | 74.3 |
| THR | 486 | TYR | 41 | 83.4 |
| TYR | 442 | ASP | 30 | 80.6 |
| GLY | 488 | LYS | 353 | 88.3 |
| SER | 432 | LEU | 45 | 81.5 |
| THR | 486 | ASN | 330 | 79.6 |
| TYR | 491 | GLY | 354 | 83.6 |
| GLN | 492 | GLN | 325 | 69.7 |
| TYR | 436 | ASP | 38 | 89.7 |
| THR | 487 | GLY | 326 | 79.9 |
| GLY | 488 | ASP | 355 | 85.8 |
| TYR | 481 | ASP | 38 | 84.6 |
| THR | 485 | ASN | 330 | 82.9 |
| TYR | 475 | PHE | 28 | 70.1 |
| ARG | 426 | GLN | 325 | 79.6 |
