# Supplementary material for: Coevolution, Dynamics and Allostery Conspire in Shaping Cooperative Binding and Signal Transmission of the SARS-CoV-2 Spike Protein with Human Angiotensin-Converting Enzyme 2
Source: Int J Mol Sci. 2020 Nov 4;21(21):8268. doi: 10.3390/ijms21218268 (PMC7672574; doi:10.3390/ijms21218268)
Supplement: Supplementary file 1 [file ijms-21-08268-s001.zip › SUPPLEMENTARY_INFORMATION/Table S5.docx]

**Table S5. The ensemble-averaged contact time of the interfacial contacts (ICs) in the SARS-CoV-2-RBD complex with ACE2 (pdb id 6M0J).**

| **SARS-CoV-2 Residue** | **Number** | **ACE2 Residue** | **Number** | **Contact time (%)** |
| --- | --- | --- | --- | --- |
| GLN | 498 | LEU | 45 | 93.5 |
| TYR | 449 | GLN | 42 | 90.3 |
| TYR | 489 | GLN | 24 | 91.2 |
| GLN | 498 | LYS | 353 | 92.3 |
| GLN | 498 | TYR | 41 | 94.5 |
| TYR | 505 | GLY | 354 | 89.5 |
| PHE | 490 | LYS | 31 | 93.2 |
| TYR | 449 | ASP | 38 | 94.6 |
| SER | 477 | GLN | 24 | 92.8 |
| TYR | 489 | TYR | 83 | 95.6 |
| GLY | 496 | ASP | 38 | 94.2 |
| GLY | 502 | GLY | 354 | 90.9 |
| TYR | 505 | ARG | 393 | 97.4 |
| TYR | 505 | LYS | 353 | 98.2 |
| TYR | 453 | HIS | 34 | 92.3 |
| PHE | 486 | TYR | 83 | 94.2 |
| GLY | 502 | ASP | 355 | 92.7 |
| LEU | 455 | LYS | 31 | 62.8 |
| LYS | 417 | ASP | 30 | 89.7 |
| ASN | 501 | ASP | 355 | 88.4 |
| TYR | 489 | LYS | 31 | 56.9 |
| TYR | 489 | THR | 27 | 89.9 |
| GLN | 498 | GLN | 42 | 90.4 |
| ASN | 501 | TYR | 41 | 95.6 |
| GLN | 493 | GLU | 35 | 87.6 |
| ASN | 487 | GLN | 24 | 84.8 |
| GLN | 493 | LYS | 31 | 94.3 |
| PHE | 486 | GLN | 24 | 90.2 |
| GLY | 446 | GLN | 42 | 89.5 |
| ALA | 475 | SER | 19 | 80.4 |
| LEU | 455 | ASP | 30 | 90.4 |
| THR | 500 | GLY | 354 | 87.6 |
| GLY | 447 | GLN | 42 | 85.9 |
| PHE | 486 | MET | 82 | 92.4 |
| PHE | 486 | LEU | 79 | 93.7 |
| LYS | 417 | HIS | 34 | 96.5 |
| ASN | 501 | LYS | 353 | 90.5 |
| ASN | 487 | TYR | 83 | 94.3 |
| LEU | 455 | HIS | 34 | 92.4 |
| ALA | 475 | GLN | 24 | 88.6 |
| PHE | 497 | LYS | 353 | 91.2 |
| ALA | 475 | THR | 27 | 92.3 |
| TYR | 489 | PHE | 28 | 86.3 |
| PHE | 456 | ASP | 30 | 92.4 |
| ASN | 487 | PHE | 28 | 94.3 |
| THR | 500 | LEU | 45 | 89.3 |
| GLY | 496 | LYS | 353 | 85.4 |
| PHE | 456 | THR | 27 | 88.2 |
| GLU | 484 | LYS | 31 | 90.4 |
| GLY | 476 | GLN | 24 | 86.7 |
| GLN | 498 | ASP | 38 | 92.5 |
| THR | 500 | ASN | 330 | 82.1 |
| PHE | 456 | LYS | 31 | 92.3 |
| GLN | 493 | HIS | 34 | 94.3 |
| TYR | 505 | GLU | 37 | 86.3 |
| GLY | 446 | LEU | 45 | 91.2 |
| GLY | 502 | LYS | 353 | 95.6 |
| THR | 500 | TYR | 41 | 96.8 |
| TYR | 495 | LYS | 353 | 90.4 |
| TYR | 505 | ALA | 386 | 84.3 |
| THR | 500 | ASP | 355 | 85.6 |
| THR | 500 | LYS | 353 | 93.3 |
| VAL | 503 | GLY | 354 | 88.6 |
| ASN | 501 | GLY | 354 | 90.4 |
| TYR | 473 | THR | 27 | 79.4 |
| THR | 500 | ARG | 357 | 81.2 |
